# Totum-63, a plant-based polyphenol-rich extract, in prediabetes or early-stage type 2 diabetes: an international, multicenter, randomized controlled trial

**DOI:** 10.1038/s41467-026-75626-0

**Published:** 2026-07-20

**Authors:** Vivien Chavanelle, Yolanda F. Otero, Murielle Fayat Cazaubiel, Jean-Marie Bard, Bruno Pereira, Véronique Sapone, Annie Bouchard-Mercier, Maxime Bargetto, Nathalie Boisseau, Bruno Guigas, Sébastien Peltier, Florian Le Joubioux, Thierry Maugard, Samy Hadjadj, Pascal Sirvent, André Marette

**Affiliations:** 1 Valbiotis R&D Center, Valbiotis, 4 rue Eric Tabarly, Périgny, France; 2 UE 2160 ISOMer-IUML FR3473 CNRS, Laboratory of General and Applied Biochemistry, Nantes University, Nantes, France; 3 CHU Clermont-Ferrand, Biostatistics Unit, MAGMA Clinical Investigation Unit, DRCI, Clermont-Ferrand, France; 4 Université Clermont Auvergne, Laboratoire des Adaptations Métaboliques à l’Exercice en conditions Physiologiques et Pathologiques (AME2P), Clermont-Ferrand, France; 5 Leiden University Center for Infectious diseases, Subdepartment Research, Systemic Immunometabolism group, Leiden University Medical Center, Leiden, The Netherlands; 6 Equipe BCBS (Biotechnologies et Chimie des Bioressources pour la Santé), La Rochelle Université, UMR CNRS 7266 LIENSs, La Rochelle, France; 7 Institut du Thorax, INSERM, CNRS, UNIV Nantes, CHU Nantes, Nantes, France; 8 Centre de recherche de l’Institut Universitaire de Cardiologie et Pneumologie de Québec (IUCPQ), Université Laval, Québec City, QC Canada; 9 Centre Nutrition, santé et société (NUTRISS), Université Laval, Québec City, QC Canada; 10 Institute of Nutrition and Functional Foods (INAF), Université Laval, Québec City, QC Canada; 11 Department of Medicine, Faculty of Medicine, Université Laval, Québec City, QC Canada

**Keywords:** Type 2 diabetes, Risk factors, Pre-diabetes

## Abstract

This multicenter, parallel-arm, superiority, randomized, double-blind, placebo-controlled study evaluates the efficacy of Totum-63 (T63), a polyphenol-rich blend of plant extracts, in improving fasting plasma glucose (FPG) in individuals with prediabetes or early-stage type 2 diabetes (T2D). Individuals from seven European countries with FPG between 110-220 mg/dL were randomized to receive placebo (PBO, n = 210), T63 (5 g/d) distributed thrice daily (T63 TID, n = 212) or twice daily (T63 BID, open-label arm, n = 214) over six months. Individuals were randomly assigned using a dynamic randomization algorithm, stratified by their glycemic status at baseline (FPG < 126 mg/dL or FPG ≥ 126) and the investigational site country. The primary outcome was the FPG change in T63 TID compared to PBO in the intention-to-treat population. Secondary assessments included changes in diabetes biomarkers, lipid profile, anthropometric measurements and subpopulation analyses based on glycemic status. During the whole study, neither the investigators nor the subjects were aware of the product they tested, except in the open arm. After six months, FPG was significantly reduced in the T63 TID group (PBO-adjusted mixed model estimated difference, PBO-AD = −3.8 mg/dL, 95% CI: [−5.9; −1.7], p = 0.0149) and the T63 BID group (PBO-AD = −5.5 mg/dL, 95% CI: [−9.7; −1.3] p < 0.001). Hence, T63 may represent a non-pharmaceutical strategy for T2D prevention. ClinicalTrials.gov Identifier: NCT04423302. Funding: This trial was funded by Valbiotis®.

## Abstract (web summary)

A large multicenter international trial shows that Totum-63, a plant-based supplement, lowers fasting blood sugar in people with prediabetes or early type 2 diabetes and improves metabolic markers, supporting its potential role in diabetes prevention

## Introduction

Diabetes has become one of the main causes of mortality worldwide, affecting 529 to 536.6 million people in 2021, with type 2 diabetes (T2D) accounting for 96% of all cases. This number is expected to rise to 1.31 billion by 2050, driven by the increasing prevalence of T2D^1,2^. Prediabetes is a metabolic state defined by hyperglycemia and/or impaired glucose tolerance remaining below T2D thresholds^3^. Although asymptomatic, individuals with prediabetes are at high risk of developing T2D^4^, multiple studies have shown that prediabetes and its progression to early-stage T2D are preventable through lifestyle modifications^3,5^. However, these interventions are undermined by poor long-term adherence, and prediabetes incidence continues to rise^6^, highlighting the urgent need for novel strategies. Totum-63 (T63) is a combination of 5 polyphenol-rich plant extracts, selected for their direct antidiabetic action or their effect against mechanistic targets related to the disease, based on the literature. Preclinical studies have revealed that T63 targets multiple pathogenic mechanisms involved in obesity and T2D. Indeed, T63 was found to enhance systemic glucose tolerance and peripheral insulin sensitivity^7,8^, reduce carbohydrate and lipid intestinal absorption^9^, along with a modulation of incretin secretion^10^ and a mild inhibition of digestive enzymes^11^ in vitro and in animal models of obesity and T2D. Furthermore, a preliminary randomized, double-blind, placebo-controlled pilot trial demonstrated that T63 improved fasting blood glucose and the glycemic response to a glucose challenge in a small cohort of 51 individuals with prediabetes or newly diagnosed T2D^12^. The objective of this first large-scale, multicenter, randomized, placebo-controlled clinical trial, was to evaluate the safety and efficacy of T63, administered twice or thrice daily, in individuals with prediabetes or newly diagnosed T2D. Notably, this study was conducted across a heterogeneous population from 7 European countries and discusses for the first time the benefits of T63 in different pathological subpopulations (individuals with prediabetes or with T2D). We show in this work that T63 supplementation improves fasting plasma glucose (FPG), HbA1c, glucose tolerance, body weight, and lipid profile in individuals with prediabetes or type 2 diabetes.

## Results

### Baseline characteristics, compliance and tolerability

**Fig. 1:**
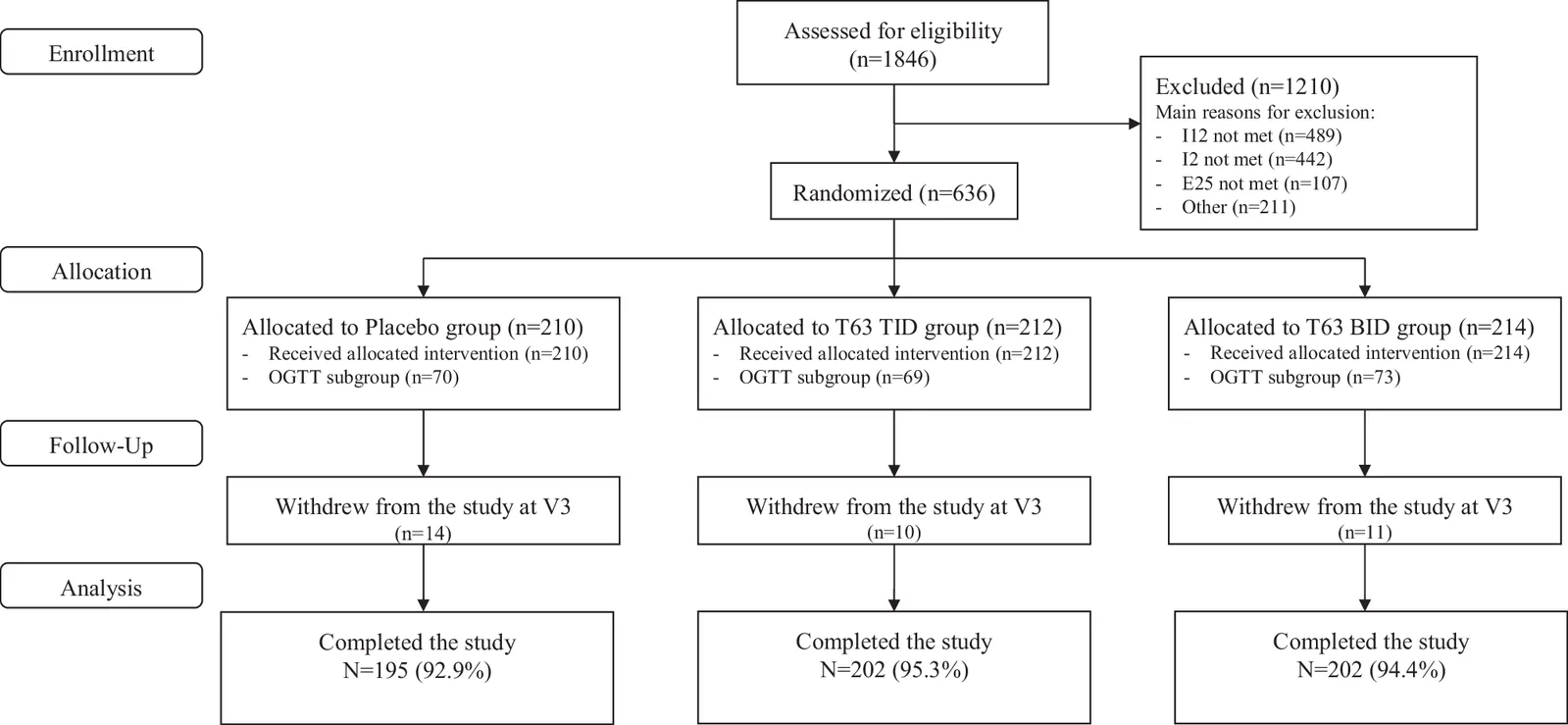
Study flow of the participants.

Fig. 1 shows the diagram flow of the distribution of the population. The trial was completed. Among 1846 screened subjects, 636 were eligible and randomly assigned to the 3 groups. 599 participants completed the study: 92.9% in the PBO group, 95.3% in the T63 TID group and 94.4% in the T63 BID group. The inclusion visits (V0) took place between July 8th, 2020, and July 26th, 2022. The last patient visit was on May 5th, 2023. Women accounted for 61.4, 53.8, and 60.3% of all participants in groups PBO, T63 TID, and T63 BID, respectively. Among the participants, 22.9, 18.9, and 22.0% had T2D (FPG ≥ 126 mg/dL) in the PBO, T63 TID, and T63 BID groups, respectively, while the remainder had prediabetes. No significant differences between groups were noted at baseline (Table 1). Global compliance was high across all groups, (97.1% in PBO, 98.0% in T63 TID and 97.4% in T63 BID, Table 2). 661 adverse events (AE, Table 3) were reported: 225 in PBO, 206 in T63 TID and 230 in T63 BID, affecting 253 participants (81 in PBO, 90 in T63 TID and 82 in T63 BID). 13 Serious AE (SAE) occurred in 10 participants (1.6%): 1 in PBO (0.5%), 6 in T63 TID (2.8%) and 3 in T63 BID (1.4%). 11 SAE were treatment-emergent, but only one had a causal relation with the study product (T63 TID) that could not be excluded (viral meningitis that started and resolved without sequelae between V2 and V3, with no product interruption).

**Table 1:** Baseline characteristics

Variable	PBO (*n* = 210)	T63 TID (*n* = 212)	T63 BID (*n* = 214)	*p*value
**Age, years**	56.8 ± 9.2	57.0 ± 8.6	55.9 ± 9.4	*0.3986*
**Sex, n (%)**				*0.2263*
** Women**	129 (61.4)	114 (53.8)	129 (60.3)	
** Men**	81 (38.6)	98 (46.2)	85 (39.7)	
**Prediabetes/T2D, n (%)**				*0.5745*
** Prediabetes**	162 (77.1)	172 (81.1)	167 (78.0)	
** T2D**	48 (22.9)	40 (18.9)	47 (22.0)	
**Body weight, kg**	93.1 ± 1.1	93.6 ± 1.2	90.4 ± 1.1	*0.0979*
**BMI, kg/m2**	32.4 ± 0.3	32.2 ± 0.3	31.8 ± 0.3	*0.2905*
**Waist circumference, cm**	109.4 ± 0.9	108.7 ± 0.9	107.4 ± 0.8	*0.2494*
**Hip circumference, cm**	113.7 ± 0.8	113.2 ± 0.8	112.1 ± 0.7	*0.3683*
**Waist/Hip ratio**	0.97 ± 0.01	0.96 ± 0.01	0.96 ± 0.01	*0.8611*
**Fasting plasma glucose, mg/dL**	122.0 ± 1.6	122.0 ± 1.5	124.4 ± 1.5	*0.4402*
**Insulin, mU/L**	18.3 ± 0.9	18.2 ± 0.8	18.9 ± 0.9	*0.8246*
**2-h OGTT glycemia, mg/dL**	149.1 ± 7.9	154.2 ± 6.5	155.7 ± 6.6	*0.7885*
**2-h OGTT insulin, mU/L**	91.8 ± 11.2	91.1 ± 11.0	87.4 ± 12.4	*0.9593*
**Triglycerides, g/L**	1.35 ± 0.06	1.33 ± 0.05	1.28 ± 0.04	*0.5749*
**LDL-C, g/L**	1.15 ± 0.02	1.16 ± 0.03	1.17 ± 0.03	*0.8679*
**Total cholesterol, g/L**	1.90 ± 0.03	1.92 ± 0.03	1.92 ± 0.03	*0.9171*
**Systolic blood pressure, mmHg**	132.6 ± 0.1	132.0 ± 0.8	132.8 ± 0.8	*0.8075*
**Diastolic blood pressure, mmHg**	81.4 ± 0.6	81.8 ± 0.6	81.0 ± 0.6	*0.6126*
**IPAQ score (MET-minutes/week)**	3965 ± 368	3879 ± 329	4029 ± 398	*0.9588*
Total energy intake (kcal/day)	1669 ± 44	1718 ± 42	1685 ± 42	*0.7106*

Data are presented as the mean ± SEM for continuous parameters or n (%) for categorical variables. Statistical comparisons were run with 1-way ANOVA. IPAQ: International Physical Activity Questionnaire. PBO: Placebo. T63 BID: Totum-63 distributed 2 times a day. T63 TID: Totum-63 distributed 3 times a day. Source data are provided as a Source Data file.

**Table 2:** Compliance

	3 months			6 months			Overall		
Groups	n	Mean	SEM	n	Mean	SEM	n	Mean	SEM
**PBO**	**210**	**97.3**	**0.5**	**210**	**96.9**	**0.5**	**210**	**97.1**	**0.4**
Prediabetes	162	97.4	0.6	162	97.0	0.5	162	97.2	0.4
T2D	48	97.2	1.1	48	96.6	1.1	48	96.8	1.0
**T63 TID**	**212**	**98.3**	**0.5**	**212**	**97.6**	**0.4**	**212**	**98.0**	**0.3**
Prediabetes	172	98.4	0.6	172	97.4	0.4	172	97.9	0.4
T2D	40	97.9	1.0	40	98.3	0.9	40	98.1	0.8
**T63 BID**	**214**	**97.3**	**0.4**	**214**	**97.2**	**0.4**	**214**	**97.4**	**0.3**
Prediabetes	167	97.6	0.4	167	97.6	0.4	167	97.8	0.3
T2D	47	96.2	0.8	47	95.6	0.7	47	96.0	0.5

Product compliance in the placebo and T63 groups. Compliance, defined as the ratio between the amount of product consumed and the expected intake, was assessed between baseline-3 months and 3–6 months (intermediate compliance) and over baseline-6 months (overall compliance). PBO: Placebo. T63 BID: Totum-63 distributed 2 times a day. T63 TID: Totum-63 distributed 3 times a day. Source data are provided as a Source Data file.

**Table 3:** Adverse events and serious adverse events

	PBO *n* = 210		T63 TID *n* = 212		T63 BID *n* = 214		
	Subjectsn (%)	Eventn	Subjectsn (%)	Eventn	Subjectsn (%)	Eventn	*p*
All AE	81 (38.6%)	225	90 (42.5%)	206	82 (38.3%)	230	*0.62*
All SAE	1 (0.5%)	1	6 (2.8%)	8	3 (1.4%)	4	*0.14*
All treatment-emergent AE	70 (33.3%)	179	77 (36.0%)	188	77 (36.0%)	188	*0.78*
All treatment-emergent SAE	1 (0.5%)	1	6 (2.8%)	7	3 (1.4%)	3	*0.14*
**Intensity of the event**							
Mild	48 (22.9%)	124	51 (24.1%)	107	53 (24.8%)	107	*0.90*
Moderate	33 (15.7%)	51	42 (19.8%)	54	42 (19.6%)	62	*0.47*
Severe	4 (1.9%)	4	10 (4.7%)	13	13 (6.1%)	19	*0.10*
Life threatening	0 (0.0%)	0	0 (0.0%)	0	0 (0.0%)	0	
Fatal	0 (0.0%)	0	0 (0.0%)	0	0 (0.0%)	0	
**Link of the AE to the research**							
Excluded	60 (28.6%)	160	65 (30.7%)	147	71 (33.2%)	171	*0.59*
Not excluded	8 (3.8%)	11	18 (8.5%)	23	11 (5.1%)	15	*0.11*
**Relation to the study product**							
Excluded	57 (27.1%)	153	64 (30.2%)	140	71 (33.2%)	160	*0.40*
Not excluded	14 (6.7%)	18	25 (11.8%)	30	16 (7.5%)	26	*0.13*
**Action taken on the study product**							
Not applicable	1 (0.5%)	1	0 (0.0%)	0	3 (1.4%)	4	*0.28*
No action	68 (32.4%)	170	73 (34.4%)	156	73 (34.1%)	173	*0.89*
Product definitively stopped	1 (0.5%)	1	6 (2.8%)	7	3 (1.4%)	3	*0.15*
Product interrupted	7 (3.3%)	7	9 (4.2%)	12	7 (3.3%)	8	*0.83*
Dosage reduced	0 (0.0%)	0	0 (0.0%)	0	0 (0.0%)	0	
**Status of the AE**							
Resolved	59 (28.1%)	149	73 (34.4%)	156	68 (31.8%)	153	*0.37*
Modification of the AE leading to a new AE	1 (0.5%)	1	1 (0.5%)	1	1 (0.5%)	1	*>0.99*
Ongoing at the end of the study	23 (11.0%)	28	16 (7.5%)	18	25 (11.7%)	33	*0.32*
Death	0 (0.0%)	0	0 (0.0%)	0	0 (0.0%)	0	

AE: Adverse event. PBO: Placebo. SAE: Serious adverse event. T63 BID: Totum-63 distributed 2 times a day. T63 TID: Totum-63 distributed 3 times a day. *P* values are from chi-squared or Fisher’s exact tests, as appropriate, comparing the three arms (global three-group comparison) for the proportion of affected subjects; tests were two-sided (α = 0.05). *P* values were not corrected for multiplicity.

### Primary outcome

**Fig. 2:**
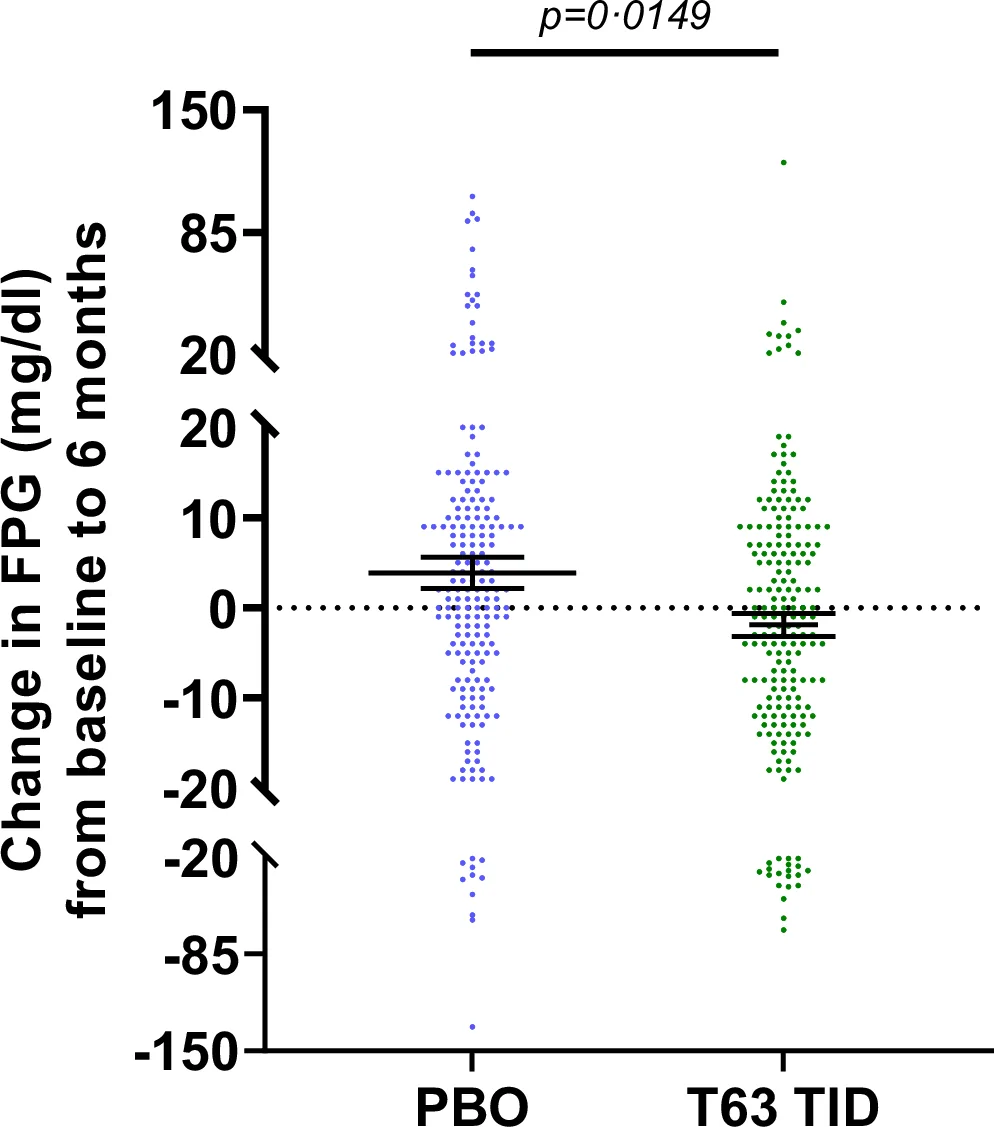
Change in FPG (mg/dL) from baseline to 6 months. Dot plot with the lines representing the mean ± SEM. Intergroup comparison was made using a linear mixed model (two-sided), considering the following fixed effects: product (TID, *n* = 198 subjects, or PBO, *n* = 204 subjects), baseline value (V1) and investigation center as random effect. PBO: Placebo. T63 TID: Totum-63 distributed 3 times a day. Source data are provided as a Source Data file.

FPG at 6 months was reduced by 1.90 ± 1.26 mg/dL in T63 TID while it increased by 3.86 ± 1.72 mg/dL in PBO, the difference was significant (PBO-AD = −3.8 mg/dL, 95% CI [−5.9; −1.7], *p* = 0.0149, Fig. 2). An additional sensitivity analysis was conducted to warrant the robustness of the findings with imputation of missing data based on the last observation carried forward. The conclusions regarding the primary outcome using this method are unchanged (*p* = 0.0124). Moreover, analysis of the primary endpoint in the per-protocol population confirmed the product effect (*p* = 0.006). Sensitivity analyses additionally accounting for potential confounders supported the robustness of the findings: statistical significance was maintained after adjustment for total energy intake (*p* = 0.027) and for the overall physical-activity score (*p* = 0.035).

### Secondary outcomes

Values at 6 months are presented in Table 4, those collected at 3 months are presented in Supplementary Table 2.

**Table 4:** Secondary analyses

		PBO			T63 TID			T63 BID		
		Total population	Prediabetes	T2D	Total population	Prediabetes	T2D	Total population	Prediabetes	T2D
**FPG (mg/dL)**		*n* = 198–209	*n* = 153–161	*n* = 45−48	*n* = 202–212	*n* = 164–172	*n* = 38–40	*n* = 203–214	n = 158-167	n = 45-47
Baseline	Mean (SEM)	122.0 (1.6)	115.2 (1.1)	145.1 (4.5)	122.0 (1.5)	115.8 (1.1)	148.4 (4.6)	124.4 (1.5)	118.6 (1.1)	145.0 (4.3)
6 months	Mean (SEM)	124.9 (2.0)*	119.2 (1.7)**	144.3 (5.9)	119.5 (1.6)	114.6 (1.4)	141.0 (5.0)	120.2 (1.7)***	115.3 (1.4)**	137.3 (5.0)*
	*P value (change 6 months-baseline, vs placebo, raw)*				***0.0042***	***0.0058***	*0.2266*	***<.0001***	***<.0001***	*0.1152*
**Fasting ins. (mU/L)**		*n* = 197–208	*n* = 152–160	*n* = 45–48	*n* = 203–212	*n* = 165–172	*n* = 37–40	*n* = 203–214	n = 158-167	n = 45-47
Baseline	Mean (SEM)	18.30 (0.87)	17.47 (0.87)	21.07 (2.39)	18.16 (0.79)	17.81 (0.86)	19.67 (2.00)	18.88 (0.94)	18.59 (1.02)	19.90 (2.28)
6 months	Mean (SEM)	18.49 (1.32)	17.82 (1.55)	20.76 (2.51)	17.38 (1.00)*	17.13 (1.14)	18.48 (2.09)	16.88 (0.80)**	16.41 (0.86)**	18.56 (1.97)
	*P value (change 6 months-baseline, vs placebo, raw)*				*0.5067*	*0.6907*	*0.4990*	*0.1899*	*0.1802*	*0.7885*
**HOMA-IR**		*n* = 197–208	*n* = 152–160	*n* = 45–48	*n* = 202–212	*n* = 164–172	*n* = 37–40	*n* = 202–214	n = 157-167	n = 45-47
Baseline	Mean (SEM)	5.60 (0.28)	5.02 (0.26)	7.53 (0.81)	5.56 (0.26)	5.19 (0.27)	7.13 (0.73)	5.83 (0.30)	5.47 (0.30)	7.09 (0.82)
6 months	Mean (SEM)	6.01 (0.56)	5.55 (0.65)	7.60 (1.02)	5.29 (0.35)*	4.98 (0.37)	6.68 (0.85)	5.11 (0.27)***	4.76 (0.26)**	6.33 (0.78)
	*P value (change 6 months-baseline, vs placebo, raw)*				*0.1608*	*0.2733*	*0.3157*	***0.0241***	***0.0331***	*0.4212*
**2-h gly. (mg/dL)**		*n* = 65–70	*n* = 54–58	*n* = 11–12	*n* = 68–69	*n* = 58–59	*n* = 10	*n* = 68–73	n = 55-60	n = 13
Baseline	Mean (SEM)	149.1 (7.9)	138.7 (7.1)	199.5 (27.8)	154.2 (6.6)	146.9 (6.8)	196.9 (17.4)	155.7 (6.4)	151.2 (6.5)	176.3 (19.7)
6 months	Mean (SEM)	153.7 (10.0)	142.7 (8.3)	207.4 (41.1)	144.5 (7.7)	133.5 (6.0)*	208.8 (33.2)	135.5 (6.2)**	132.1 (5.8)*	149.8 (21.6)
	*P value (change 6 months-baseline, vs placebo, raw)*				*0.0855*	*0.0737*	*0.8607*	***0.0170***	***0.0479***	*0.1671*
**HbA1c (%)**		*n* = 173–186	*n* = 136–145	*n* = 37–41	*n* = 183–194	*n* = 147–156	*n* = 35–38	*n* = 186–197	n = 145-154	n = 41-43
Baseline	Mean (SEM)	6.10 (0.06)	5.87 (0.04)	6.90 (0.21)	6.09 (0.06)	5.90 (0.05)	6.90 (0.18)	6.14 (0.06)	5.93 (0.04)	6.86 (0.20)
6 months	Mean (SEM)	6.06 (0.06)	5.91 (0.04)	6.58 (0.18)	5.99 (0.05)*	5.86 (0.05)	6.53 (0.16)*	5.97 (0.05)***	5.83 (0.04)*	6.48 (0.14)**
	*P value (change 6 months-baseline, vs placebo, raw)*				*0.0663*	*0.1179*	*0.2529*	***0.0033***	***0.0090***	*0.1295*
**Body weight (kg)**		*n* = 198–210	*n* = 153–162	*n* = 45–48	*n* = 205–212	*n* = 167–172	*n* = 38–40	*n* = 204–214	n = 159-167	n = 45-47
Baseline	Mean (SEM)	93.04 (1.10)	92.96 (1.26)	93.29 (2.22)	93.55 (1.18)	92.67 (1.28)	97.30 (2.92)	90.36 (1.10)	89.33 (1.20)	94.03 (2.57)
6 months	Mean (SEM)	92.15 (1.16)**	92.33 (1.34)*	91.55 (2.38)	92.60 (1.17)****	91.85 (1.26)***	95.92 (3.01)****	88.93 (1.15)****	87.92 (1.25)****	92.49 (2.72)**
	*P value (change 6 months-baseline, vs placebo, raw)*				*0.0606*	*0.2718*	***0.0182***	***0.0043***	***0.0071***	*0.3095*
**WC (cm)**		*n* = 198–210	*n* = 153–162	*n* = 45–48	*n* = 204–212	*n* = 166–172	*n* = 38–40	*n* = 203–214	n = 158-167	n = 45-47
Baseline	Mean (SEM)	109.4 (0.9)	109.2 (1.0)	110.2 (1.6)	108.7 (0.9)	107.6 (0.9)	113.5 (2.4)	107.4 (0.8)	106.8 (0.9)	109.7 (1.8)
6 months	Mean (SEM)	107.7 (0.9)****	107.5 (1.0)****	108.4 (1.8)*	107.5 (0.9)****	106.3 (0.9)****	112.4 (2.4)**	105.6 (0.9)****	105.0 (0.9)****	107.8 (1.9)****
	*P value (change 6 months-baseline, vs placebo, raw)*				*0.8562*	*0.8946*	*0.8646*	*0.1250*	*0.2268*	*0.3248*
**TG (g/L)**		*n* = 198–209	*n* = 153–161	*n* = 45–48	*n* = 204–212	*n* = 166–172	*n* = 38–40	*n* = 202–212	n = 158-166	n = 44-46
Baseline	Mean (SEM)	1.35 (0.06)	1.33 (0.07)	1.42 (0.11)	1.33 (0.05)	1.28 (0.06)	1.52 (0.10)	1.28 (0.04)	1.25 (0.04)	1.40 (0.11)
6 months	Mean (SEM)	1.39 (0.05)	1.36 (0.06)	1.51 (0.12)	1.20 (0.04)***	1.19 (0.05)*	1.27 (0.09)**	1.26 (0.05)	1.19 (0.05)*	1.49 (0.11)
	*P value (change 6 months-baseline, vs placebo, raw)*				***0.0011***	***0.0388***	***0.0019***	*0.0590*	*0.0647*	*0.6319*
**TC (g/L)**		*n* = 198–209	*n* = 153–161	*n* = 45–48	*n* = 204–212	*n* = 166–172	*n* = 38–40	*n* = 203–213	n = 159-167	n = 44-46
Baseline	Mean (SEM)	1.90 (0.03)	1.91 (0.03)	1.88 (0.06)	1.92 (0.03)	1.92 (0.03)	1.93 (0.08)	1.92 (0.03)	1.93 (0.03)	1.87 (0.06)
6 months	Mean (SEM)	1.96 (0.03)*	1.97 (0.03)	1.92 (0.06)	1.86 (0.03)*	1.87 (0.03)	1.83 (0.08)	1.90 (0.10)	1.89 (0.03)	1.97 (0.06)*
	*P value (change 6 months-baseline, vs placebo, raw)*				***0.0012***	***0.0078***	*0.0539*	***0.0389***	***0.0087***	*0.6292*
**LDL-C (g/L)**		*n* = 191–204	*n* = 148–158	*n* = 43–46	*n* = 199–210	*n* = 161–170	*n* = 37–40	*n* = 198–210	n = 155-165	n = 43-45
Baseline	Mean (SEM)	1.15 (0.02)	1.15 (0.03)	1.14 (0.05)	1.16 (0.03)	1.16 (0.03)	1.14 (0.06)	1.17 (0.03)	1.19 (0.03)	1.10 (0.06)
6 months	Mean (SEM)	1.17 (0.03)	1.18 (0.03)	1.14 (0.05)	1.14 (0.02)*	1.13 (0.03)	1.17 (0.05)	1.11 (0.03)	1.12 (0.03)*	1.09 (0.07)
	*P value (change 6 months-baseline, vs placebo, raw)*				***0.0218***	*0.0699*	*0.1563*	*0.0880*	***0.0300***	*0.6931*

Values are presented as mean (SEM). Comparisons were made using a linear mixed model on log-transformed values considering baseline (V1), 3 months (V2) and 6-months (V3) values. The number of subjects, denoted as n, is presented as an interval representing the minimum and maximum number of participants for each parameter, accounting for variations across visits (V1 to V3) due to missing values. Intergroup comparisons versus placebo were performed on the baseline-adjusted 6-month change (Δ6Months-baseline); *p* values < 0.05 are reported in bold. Intragroup comparisons versus baseline (within-group changes) were obtained as 6-month-versus-baseline contrasts from the same mixed model; they are indicated by asterisks (*p < 0.05, **p < 0.01, ***p < 0.001, ****p < 0.0001). Statistical tests were two-sided with type-I error set at 5%. Fasting ins.: fasting insulin. PBO: Placebo. T63 BID: Totum-63 distributed 2 times a day. T63 TID: Totum-63 distributed 3 times a day. WC: waist circumference. 2-h gly.: 2-h glycemia following oral glucose tolerance test. Source data are provided as a Source Data file.

### Glucose control

FPG change after 6 months was significantly different in T63 BID, compared to PBO, in total population (–4.25 ± 1.19 vs. +3.86 ± 1.72 mg/dL, PBO-AD = –5.5 mg/dL, 95% CI [–9.7; –1.3], *p* < 0.001). Subpopulation analysis revealed that FPG change after 6 months was significantly reduced in T63 TID and T63 BID in individuals with prediabetes, vs. PBO (–1.09 ± 1.26 and –3.42 ± 1.16 vs. +4.20 ± 1.69 mg/dL, PBO-AD = –3.7 mg/dL, 95% CI [–5.7; –1.7], *p* = 0.006 and PBO-AD = –5.2 mg/dL, 95% CI [–9.4; –1.1], *p* < 0.001, respectively). The reduction of FPG in the T2D subgroup (limited to 38–48 participants per arm) did not reach significance threshold for T63 TID and T63 BID, vs. PBO (–5.42 ± 1.26 and –7.13 ± 4.21 vs. +2.69 ± 5.01 mg/dL, PBO-AD = –4.3 mg/dL, 95% CI [–10.6; 2.1], *p* = 0.227 and PBO-AD = –7.6 mg/dL, 95% CI [–19.7; 4.6], *p* = 0.115, respectively).

The intergroup analysis showed no significant effect on fasting insulin, vs PBO, although fasting insulin levels were significantly reduced at 6 months in T63 TID and T63 BID vs. baseline, in the total population (Intragroup analysis, –0.83 ± 0.93 and –1.63 ± 0.72 mU/L, *p* = 0.045 and *p* = 0.004, respectively. Not significant in PBO group, +0.10 ± 1.22 mU/L, *p* = 0.296).

HOMA-IR was significantly decreased after 6 months in T63 BID vs PBO in the total population (–0.56 ± 0.21 vs. +0.48 ± 0.53, *p* = 0.024) and in participants with prediabetes (–0.66 ± 0.24 vs. +0.46 ± 0.66, *p* = 0.033). In T63 TID, only intragroup comparison of HOMA-IR at 6 months vs. baseline was significant in total population (–0.27 ± 0.32, *p* = 0.022. Not significantly altered in the PBO group: +0.48 ± 0.53, *p* = 0.774).

In the OGTT subgroup, we found a significant difference in the change in 2-h glycemia in the T63 BID group, vs PBO, in total population (–13.94 ± 6.55 vs. +7.94 ± 8.68 mg/dL, *p* = 0.017) and in participants with prediabetes (–10.96 ± 6.84 vs. +5.59 ± 8.70 mg/dL, *p* = 0.048). The changes in the T63 TID group did not reach significance threshold, vs. PBO, for total population (–9.81 ± 7.34 vs. +7.94 ± 8.68 mg/dL, *p* = 0.086) or participants with prediabetes (–13.55 ± 8.00 vs. +5.59 ± 8.70 mg/dL, *p* = 0.074), however, intragroup analysis in participants with prediabetes revealed a significant decrease in 2-h glycemia at 6 months vs. baseline, in T63 TID (–13.55 ± 8.00 mg/dL, *p* = 0.046. Not significant in PBO group, +5.59 ± 8.70 mg/dL, *p* = 0.584).

Finally, HbA1c levels were significantly reduced in the T63 BID group vs. PBO in the total population (–0.15 ± 0.06 vs. +0.03 ± 0.05%, *p* = 0.003) and in participants with prediabetes (–0.07 ± 0.04 vs. +0.05 ± 0.04%, *p* = 0.009). The intragroup analysis in participants with T2D showed a significant difference at 6 months compared to baseline in this group (–0.45 ± 0.22%, *p* = 0.005. Not significant in PBO group –0.07 ± 1.24%, *p* = 0.523). In the T63 TID group, only intragroup differences were significant, with a reduction of HbA1c in the total population at 6 months vs. baseline (–0.08 ± 0.05%, *p* = 0.038) and in the T2D subpopulation (–0.31 ± 0.13%, *p* = 0.027).

### Anthropometric measurements

BW change was significantly different in the T63 BID group vs. PBO, in the total population (–1.27 ± 0.25 vs. –0.49 ± 0.30 kg, *p* = 0.004) and in participants with prediabetes (–1.29 ± 0.29 vs. 0.46 ± 0.36 kg, *p* = 0.007). BW change was significantly different in the T63 TID group in the subpopulation with T2D, vs. PBO (–2.14 ± 0.49 vs. –0.61 ± 0.49 kg, *p* = 0.018), while it did not reach statistical significance threshold for the total population, vs. PBO (–1.17 ± 0.24 vs. –0.49 ± 0.30 kg, *p* = 0.061). No significant differences were observed in WC change among groups.

### Blood lipids

Changes in fasting TG levels were significantly different in the T63 TID group vs. PBO, in the total population –0.12 ± 0.05 vs. +0.05 ± 0.06 g/L, *p* = 0.001 as well as in participants with prediabetes (–0.10 ± 0.05 vs. +0.02 ± 0.07 g/L, *p* = 0.039) and with T2D (-0.24 ± 0.09 vs. +0.13 ± 0.10 g/L, p = 0.002). Changes in the T63 BID group did not reach statistical significance threshold, vs. PBO (–0.02 ± 0.04 vs. +0.05 ± 0.06 g/L, *p* = 0.059 in the total population, -0.04 ± 0.04 g/L vs. +0.02 ± 0.07 g/L, *p* = 0.065 in participants with prediabetes, and +0.08 ± 0.11 g/L vs. +0.13 ± 0.10 g/L, *p* = 0.632 in population with T2D). However, in this group, a significant reduction in the intragroup analysis was noted for the participants with prediabetes, at 6 months vs. baseline (–0.04 ± 0.04 g/L, *p* = 0.036). Additionally, significant changes in TC were observed in both the T63 TID and T63 BID groups compared to PBO, in the total population (–0.05 ± 0.03 and –0.01 ± 0.03 vs. +0.06 ± 0.03 g/L, *p* = 0.001 and *p* = 0.039, respectively) and in the subpopulation with prediabetes (–0.05 ± 0.03 and –0.05 ± 0.03 vs. +0.06 ± 0.03 g/L, *p* = 0.008 and *p* = 0.009, respectively). Finally, we observed significant changes in LDL-C in the T63 TID group, in the total population, vs. PBO (–0.04 ± 0.02 vs. +0.03 ± 0.02 g/L, *p* = 0.022). The difference was significant in T63 BID group in participants with prediabetes, vs. PBO (–0.06 ± 0.02 vs. +0.02 ± 0.02 g/L, *p* = 0.030; total population: –0.03 ± 0.02 vs. +0.03 ± 0.02 g/L, *p* = 0.088).

### Potential confounders

Physical activity and dietary intake were assessed using questionnaires and included as covariates in the sensitivity analyses. At baseline, total energy intake and the overall physical-activity score were comparable across groups, with no statistically significant differences (Table 1). These parameters did not change differentially between groups over the course of the intervention (Supplementary Table 3). More detailed analyses of dietary variables (percentage of energy from fat, carbohydrate, and protein; saturated-fat intake; alcohol intake) and of physical activity (type, volume, and intensity) likewise revealed no between-group differences, either at baseline or in their trajectories at 3 and 6 months (Supplementary Tables 6 and 7).

### Exploratory outcomes

#### Changes in glycemic status

**Fig. 3:**
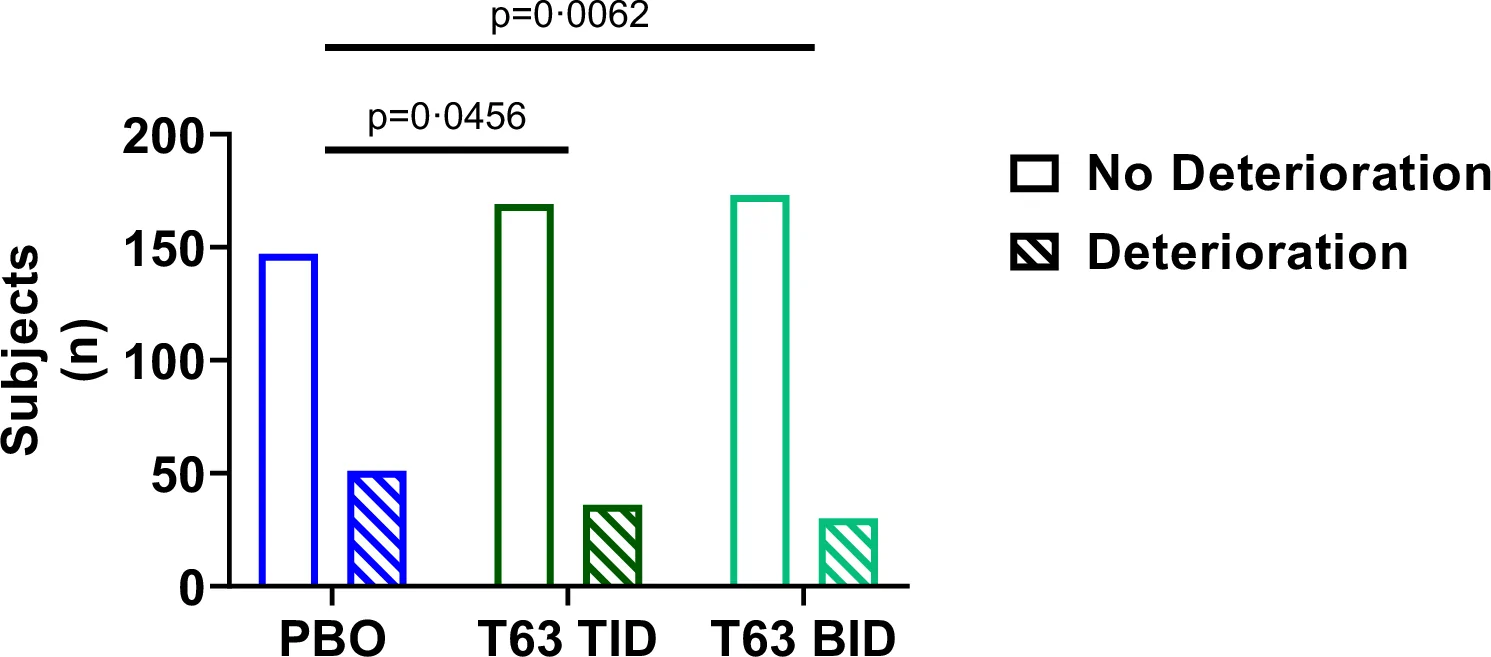
Change in glycemic status from baseline to 6 months. Deterioration or no deterioration of glycemic status in total population, as defined by the following FPG categories: 1/ FPG < 110 mg/dL; 2/ 110 < FPG < 126 mg/dL; 3/ 126 ≤ FPG < 220 mg/dl; 4/ FPG ≥ 220 mg/dl. Overall Chi2 test (*p* = 0.015) followed by pair-wise intergroup Chi2 tests (two-sided). PBO: Placebo. T63 BID: Totum-63 distributed 2 times a day. T63 TID: Totum-63 distributed 3 times a day. Source data are provided as a Source Data file.

The change in glycemic status was significantly different among groups *p* = 0.015, Fig. 3 and pairwise analysis revealed a significant difference between deterioration and no deterioration in the PBO group (respectively 51 participants, 25.8% and 147 participants, 74.2%) compared to T63 TID (respectively 36 participants, 17.6% and 169 participants, 82.4%, *p* = 0.0456), and compared to T63 BID (respectively 30 participants, 14.8% and 173 participants, 85.2%, *p* = 0.0062).

#### Linear regression of HbA1c changes as a function of baseline HbA1c

A negative correlation was observed in all 3 groups, PBO, T63 TID, and T63 BID (*p* = 0.0073, *p* < 0.0001 and *p* < 0.0001, respectively, Supplementary Fig. 1) with respective Spearman correlation coefficients of *r* = –0.3094, *r* = –0.3543, and *r* = –0.2034.

## Discussion

This is the first large-scale trial assessing the effects of T63 on FPG over a 6-month supplementation period (5 g/day) in individuals with prediabetes or untreated T2D. In the double-blind, placebo-controlled arm, T63 TID significantly reduced FPG by 1.90 ± 1.26 mg/dL (PBO-AD = –3.8 mg/dL, 95% CI [–5.9; –1.7]). Additionally, T63 BID (open-label) also demonstrated a significant FPG reduction of 4.25 ± 1.29 mg/dL, (PBO-AD = –5.5 mg/dL, 95% CI [–9.7; –1.3]). T63 showed good compliance, was well tolerated, and no differences in AE were observed vs. PBO.

The magnitude of the decrease in FPG aligns with findings from a previous pilot study using T63 at the same dose and distribution pattern (5 g/day, TID) in a similar population, reporting a reduction of 4.32 mg/dL in FPG after 6 months, or a PBO-corrected difference of 12.97 mg/dL^12^. Interestingly, we demonstrated here that the effects were particularly significant in participants with prediabetes, with FPG reductions of 1.09 ± 1.26 mg/dL (PBO-AD = –3.7 mg/dL, 95% CI [–5.7; –1.7]) and 3.42 ± 1.16 mg/dL (PBO-AD = –5.2 mg/dL, 95% CI [–9.4; –1.1]) observed in the T63 TID and T63 BID groups, respectively. These results are comparable to those obtained in the Diabetes Prevention Program study achieved using lifestyle modification (diet, exercise) or metformin for 6 months^13^, reporting -4 mg/dL (median) for both interventions, compared to –1 mg/dL in the control group. Recently, concurring additional evidences were introduced in a meta-analysis demonstrating average FPG reductions of 4.7 mg/dL with exercise training and 5.9 mg/dL with metformin in prediabetes based on per-protocol (PP) analyses. When restricted to intention-to-treat (ITT) studies, the corresponding reductions were 2.3 mg/dL and 3.1 mg/dL, respectively^14^.

These results are especially noteworthy since a previous retrospective cohort study of real-world patients has reported that the risk of developing T2D increases by 7–8% for each 1 mg/dL rise in fasting glycemia, in this high-risk subpopulation^15^. In practical terms, for the entire population in this trial, we demonstrated that these improvements led to a significantly smaller number of participants experiencing a deterioration in their glycemic status. T63 may thereby reduce the risk of progression toward more severe glycemic dysregulation, in individuals at high risk of developing T2D or newly diagnosed T2D patients. In line with this, recent reviews have concluded that early interventions in individuals with prediabetes can substantially reduce the likelihood of progression to T2D, as even modest reductions in FPG have shown long-term benefits^4,16^. The observed effects on fasting plasma glucose in the subgroup with early T2D (placebo-adjusted mean changes of –4.3 mg/dL in the T63 TID group and –7.6 mg/dL in the T63 BID group) should be interpreted with caution given the more limited sample size (approximately 40–45 participants per group). Nonetheless, comparison with the recent meta-analysis by Zhao et al. indicates that the magnitude of effect remains comparable to that of exercise interventions^14^. In contrast, pharmacological treatments such as metformin appear to exert stronger effects than either exercise or the results obtained with T63 in this early-stage T2D population^14,17^.

Other markers of glucose control, such as HbA1c and 2-h glucose response following an OGTT, were also improved after 6 months of T63 supplementation, although the difference vs. PBO group did not reach statistical threshold in the T63 TID group (*p* = 0.0663 and *p* = 0.0855, respectively). In parallel, the analysis of HbA1c changes after 6 months (compared to baseline) as a function of baseline HbA1c indicates that higher baseline HbA1c levels were associated with greater reductions thereof. In line with this, HbA1c at 6 months was significantly reduced in both T63 supplemented groups (intragroup analysis, vs. baseline) in participants with T2D. Elevated HbA1c is a surrogate marker of long-term dysregulated glucose metabolism, characteristic of individuals with a greater degree of glycemic imbalance who are typically at higher risk for the progression to T2D and related complications, such as cardiovascular diseases^18^. These findings therefore suggest that patients with elevated HbA1c are likely to experience substantial improvements with T63 supplementation.

In addition to that, we noted a significant reduction in BW, compared to PBO, in the T63 BID group in the total population. Although intergroup comparison did not reach statistical significance threshold in the T63 TID group (*p* = 0.0606) in the total population, a significant change was observed in this group in the T2D subpopulation. These findings are meaningful, as a 16% reduction in T2D risk per kilogram of BW lost was reported in the Diabetes Prevention Program cohort^19^. In accordance with this, major health organizations recommend weight loss (starting from 5%) as a key goal for individuals with prediabetes or T2D, highlighting its benefits for improving glycemic control and reducing medication requirements^20,21^.

Additionally, exploratory analyses showed that T63 supplementation elicited beneficial changes in lipid profile, including significant improvements in circulating TG, TC, and LDL-C. While modest, these improvements may nonetheless be beneficial, as elevated circulating lipids are associated with an increased risk of T2D^22^. However, since participants did not have dyslipidemia at baseline in this trial, the clinical significance of these findings must be consolidated in a cohort with abnormal circulating lipid levels. Interestingly, intergroup comparisons revealed that these effects were more significant in the T63 TID group than in the T63 BID group. While the present trial was not designed to investigate this particular aspect, it can be speculated that this difference could be linked to certain effects of T63 previously reported in animal models. Indeed, we observed a reduction in circulating TG following an acute lipid tolerance test when a single dose of T63 was administered beforehand to mice fed a high fat diet^9^. If this mechanism holds in humans, T63 may thus provide greater benefits on circulating lipid profile when taken before each meal (breakfast, lunch, and dinner, TID setup), compared to only before breakfast and dinner (BID setup).

One limitation of this study is that, while the results obtained in the T63 TID group were derived from an optimal randomized, placebo-controlled, double-blind trial setup, those from the T63 BID group were generated in an open-label arm and should therefore be interpreted with caution. Although standardized biochemical markers (FPG, 2-h OGTT glucose, and HbA1c) were used to assess glycemic control, continuous glucose monitoring (CGM) could have provided complementary information on short-term glycemic variability and postprandial dynamics, which were not captured in the present study.

In conclusion, T63, a polyphenol-rich combination of 5 plant extracts, demonstrated efficacy in reducing FPG in individuals with prediabetes or newly diagnosed T2D when administered over six months (5 g/day). The product was well tolerated and showed additional benefits in improving HbA1c, circulating lipid profile, or decreasing BW. Moreover, the deterioration of glycemic status was significantly reduced by T63 supplementation. Together, these findings support the potential role of T63 in the prevention and management of T2D.

## Methods

### Study design

The study protocol complies with all relevant ethical regulations and was registered at Clinicaltrials.gov (NCT04423302, the study protocol is available online at https://clinicaltrials.gov/study/NCT04423302). This trial (REVERSE-IT) was a multicenter, randomized, double-blind, placebo-controlled study with three parallel groups: two double-blind arms and one open-label arm. It was carried out in 52 sites across 7 European countries between 2020 and 2023. All patients gave written informed consent.

### Institutional review boards

The study protocol and related documents were approved by the following ethics committees: Ethics Committee “Comité de Protection des Personnes” (CPP) Sud-Méditerranée IV, Montpellier, France (Ref. 20 05 04 / SI 20.00162.200504); Ethics Committee -State Chamber of Physicians of Baden-Württemberg, Stuttgart, Germany (Ref. F-2020-124); Ethics Committee of the Bavarian State Chamber of Physicians, Munich, Germany (Ref. PEC16075 - mb20061); Ethics Committee of the Saxon State Chamber of Physicians, Schützenhöhe, Dresden, Germany (Ref. EK-BR-1/21-1); Ethics Committee, Chamber of Physicians of Saarland, Saarbrücken, Germany (Ref. Ha 258/20); Ethics Committee at Universitätsmedizin Rostock, Rostock, Germany (Ref. A 2020-0289); Ethics Committee of the Chamber of Physicians of Hamburg, Hamburg, Germany (Ref. 2021-10398-BO-bet); Ethics Committee of the Chamber of Physicians of Nordrhein, Düsseldorf, Germany (Ref. 2020411); Ethics Committee of the Chamber of Physicians of Niedersachsen, Hannover, Germany (Ref. Grae/010/2021); Central Area Section Ethics Committee, Calabria Region, Catanzaro, Italy (Ref. No. 80 of 18 March 2021); Ethics Committee of Sapienza University, Roma, Italy (Ref. 6373); Ethics Committee of the San Raffaele Hospital, Scientific Institute for Research, Hospitalization and Healthcare, Milano, Italy (Ref. 100/2021); Ethics Committee Azienda Ospedaliera Universitaria Policlinico Paolo Giaccone, Palermo, Italy (Ref. 150109 Min. No. 02/2021); Local Ethics Committee at DKC Aleksandrovska EOOD, Sofia, Bulgaria (Ref. 439/17.08.2021); Ethics Committee at UMBAL Aleksandrovska, Sofia, Bulgaria (Ref. 143/10.09.2021); Ethics Committee at Diagnostic Consultative Center XX-Sofia EOOD, Sofia, Bulgaria (Ref. 130/14.05.2021); Ethics Committee at 4th MHAT - Sofia EAD, Sofia, Bulgaria (Ref. 1/08.07.2021); MHAT Sveta Karidad EAD, 23 A Nikola Vaptsarov Str., Plovdiv, Bulgaria (Ref. 20.12.2021); Diagnostic Consultative Center VII District Yuzhen, Plovdiv, Bulgaria (Ref. 393/23.12.2021); AIPSIMP-EBO “Endo-Med Consult” EOOD, Plovdiv, Bulgaria (Ref. 3/20.12.2021); MBAL Botevgrad EOOD, Botevgrad, Bulgaria (Ref. 296/07.12.2021); Bioethics Committee at the District Medical Chamber in Kraków, Kraków, Poland (Ref. 366/KBL/OIL/2021); Ethics Committee, Bucharest City Hall / Municipal Health Authority, “Sf. Luca” Chronic Diseases Hospital, Bucarest, Romania (Ref. 539/17.01.2022); Scientific & Research Ethics Committee of the Medical Research Council (ETT TUKEB), Budapest, Hungary (Ref. 30090-10/2021/EÜIG).

### Eligibility

To be eligible to the study, male and female subjects will have to fulfill the following criteria (Assessment based on the medical examination performed at V0 with a checking at V1 when applicable): I1. Age between 18 and 70 years (including ranges); I2. Dysglycemic, prediabetic, or newly diagnosed type 2 diabetic, without any clinical symptoms of diabetes (e.g., polyuria, polydipsia, blurred vision…) and not requiring immediate anti-diabetic treatment; I3. Body mass index (BMI) between 25 and 40 kg/m² (including ranges); I4. Waist circumference >102 cm for men and > 88 cm for women (–2% margin allowed, respectively ≥100 cm and ≥86.5 cm); I5. Weight stable within ±5% in the last three months; I6. No significant change in food habits or in physical activity in the 3 months before the randomization and agreeing to follow hygiene and dietary (HD) recommendations given during the study; I7. For women: non-menopausal with the same reliable contraception since at least three months before the beginning of the study and agreeing to keep it during the entire duration of the study (hormonal contraception, intra-uterine device, or surgical intervention) or menopausal with or without hormone replacement therapy (estrogenic replacement therapy begun from less than 3 months excluded); I8. Good general and mental health according to the opinion of the investigator: no clinically significant and relevant abnormalities of medical history or physical examination; I9. Able and willing to participate to the study by complying with the protocol procedures as evidenced by his dated and signed informed consent form; I10. Affiliated to a social security scheme; I11. Agreeing to be registered on the volunteers in biomedical research (applicable only for French centers). At V0 biological analysis, the subjects will be eligible to the study on the following criteria: I12. Fasting plasma glucose concentration ≥110 mg/dL; A re-screening can occur from 4 weeks after the exit of study for failure to comply with the one or more of the eligibility criteria listed above.

Subjects with the following criteria will be considered as non-eligible to the study (assessment based on the medical examination performed at V0 with a checking at V1 when applicable): E1. Suffering from a metabolic disorder such as treated diabetes, uncontrolled thyroidal trouble or other metabolic disorder needing a dose adjustment in drug intervention according to the professional recommendations; E2. Suffering from an uncontrolled arterial hypertension (systolic blood pressure ≥160 mmHg and/or diastolic blood pressure ≥100 mmHg); E3. With a history of retinopathy, ischemic cardiovascular event, having undergone recent surgical procedure in the past 6 months or in the 9 months to come; E4. Suffering from a severe chronic disease (e.g., cancer, HIV, renal failure, hepatic or biliary disorders, ongoing, chronic inflammatory digestive disease, arthritis, or other chronic respiratory trouble, etc.) or gastrointestinal disorders found to be inconsistent with the conduct of the study by the investigator (e.g., celiac disease); E5. Under antidiabetic drug (e.g. biguanides, sulfonylureas, glinides, gliptines, glitazones, gliflozines, α-glucosidase inhibitors, incretins and insulin); E6. Under lipid-lowering treatment (e.g., statins, fibrates, ezetimibe, bile acid sequestrants, niacin, etc.) for less than 3 months or modification of the treatment dose for less than 3 months before the randomization. Subjects with a stable lipid-lowering treatment since at least three months can be included in the study; E7. Under medication which could affect glucose and/or lipid homeostasis parameters or stopped less than 3 months before randomization (e.g. beta 2 agonists like salbutamol, Angiotensin Converting Enzyme (ACE) inhibitors, beta blockers, thiazide diuretics, Selective Serotonin Reuptake Inhibitors (SSRIs), Mono-Amine Oxidase Inhibitors (MAOIs), neuroleptics, long-term corticosteroid systemic drugs, systemic antibodies, androgens, phenytoin, interferons, immunosuppressants, antivirals and antiretrovirals, etc.): - Beta 2 agonists like salbutamol, ACE inhibitors, beta blockers, thiazide diuretics, SSIRs, MAOIs are tolerated only if stable since more than 3 months before the randomization and maintained during the whole study; - The others drugs (neuroleptics, long-term corticosteroid systemic drugs, systemic antibodies, androgens, phenytoin, interferons, immunosuppressants, antivirals and antiretrovirals, etc.) are not allowed during the study; E8. Regular intake of dietary supplements or “health foods” or products rich in plant stanol or sterol (like PRO-ACTIV® or DANACOL® products), rich in long chain omega-3 fatty acids (especially soft gels containing fish oils), or in other substances intended to reduce glycemia (e.g. beta-glucans, konjac, olive leaf extract, berberine, cinnamon, etc.) or stopped less than 3 months before the randomization; E9. Under treatment or dietary supplement which could significantly affect parameter(s) followed during the study according to the investigator or stopped in a too short period before the randomization (for example consumed in the month before randomization); E10. With a known or suspected food allergy or intolerance or hypersensitivity to any of the study products’ ingredient; E11. Consuming more than 3 standard drinks daily of alcoholic beverage for men or 2 standard drinks daily for women; E12. With extreme and/or unbalanced eating habits (e.g. vegetarian, vegan, skipping meals regularly); E13. With a personal history of anorexia nervosa, bulimia, or significant eating disorders according to the investigator; E14. Smoking more than 20 cigarettes daily. The subject should be able not to smoke during the visits (maximum 4 h); E15. Having a lifestyle deemed incompatible with the study according to the investigator including high level of physical activity (defined as more than 10 h of intense physical activity a week, walking excluded); E16. Pregnant (as evidenced by a positive test for β-HCG, i.e.,> 5 mUI/mL, realized at V0) or lactating women or intending to become pregnant within 9 months ahead; E17. Who made a blood donation in the 3 months before the randomization or intending to make it within 9 months ahead; E18. Taking part in another clinical trial or being in the exclusion period of a previous clinical trial; E19. Having received, during the last 12 months, indemnities for clinical trial higher or equal to 4500 Euros (applicable only for French centers); E20. Under legal protection (guardianship, wardship) or deprived from his rights following administrative or judicial decision; E21. Presenting a psychological or linguistic incapability to sign the informed consent; E22. Impossible to contact in case of emergency; E23. Any condition assessed by the investigator which could endanger patient’s safety or the conduct of the study (e.g. device related contraindication for impedancemetry and/or DEXA such as pacemaker or electronic implant).

At V0 biological analysis, the subjects will be considered as non-eligible to the study on the following criteria:

E24. Fasting glucose plasma concentration >220 mg/dL; E25. Fasting blood triglycerides >2.2 g/L; E26. TSH outside the laboratory normal values; E27. Fasting blood LDL cholesterol >1.9 g/L or non-HDLc>2.2 g/L or any condition requiring a therapeutic dose adjustment during the trial according to the professional recommendations; E28. Blood AST, ALT, or GGT >3xULN (laboratory Upper Limit of Normal); E29. Blood creatinine concentration >125 μmol/L; E30. eGFR (calculated by CKD-EPI formula) <60 mL/min/1.73 m²; E31. Complete blood count (CBC) with hemoglobin <11 g/dL or leukocytes <3000 /mm3 or leukocytes >16,000 /mm3 or clinically significant abnormality according to the investigator.

A re-screening can occur from 4 weeks after the exit of study for failure to comply with the one or more of the eligibility criteria listed above.

The list of the eligibility and exclusion criteria is available in Supplementary Table 1. No major changes in methods were implemented after study commencement.

### Procedures

Patients who provided consent were assessed for eligibility (V0). 1 to 4 weeks after, eligible volunteers were selected to undergo baseline blood sampling and anthropometric measurements (V1). 636 participants were then randomly assigned (1:1:1) to receive either T63 (5 g/day, TID: three times a day, 8 capsules split across morning (3) lunch (2) and dinner (3)), placebo (PBO) (TID, same regimen), or T63 (5 g/day, BID: twice a day, 8 capsules split across morning and dinner). Biofortis (Saint-Herblain, France) was responsible for the enrollment, allocation, and follow-up of the participants. The TID groups were double-blind while the BID regimen was open-label. The BID regimen was conducted open-label as an exploratory arm because maintaining blinding would have required an additional BID-placebo group and a substantially more complex, quasi-separate study. For the double-blind arms (T63 TID and placebo), participants, investigators, care providers, and outcome assessors were blinded to treatment allocation throughout the study. Blinding was ensured by the use of identical capsules in terms of labeling, appearance, odor, and taste. Study products were packaged in identical opaque capsules, and no treatment-related differences (e.g., urine color or stool appearance) were observed that could have compromised blinding. Group allocation was generated using a dynamic randomization algorithm, designed to minimize imbalance between the 3 arms within the strata defined by two factors: 1/ Risk level at baseline (subject with FPG < 126 mg/dL at V0 or V1, subject with FPG ≥ 126 mg/dL at V0 and V1), with ratio of 60:40 for prediabetes and T2D; 2/ Investigational site country (France, Germany, Italy, Bulgaria, Hungary, Poland and Romania). Patients were instructed to take the assigned product for 24 weeks. General lifestyle recommendations were also provided (Supplementary methods). Blood was drawn from 12-h fasted participants at the beginning of the study (V1), after 12 weeks ( ± 5 days, V2), and at the end of the study (24 weeks ± 5 days, V3) for biochemical measurements. FPG was assessed by an enzymatic colorimetry assay with hexokinase (Kit GLUC3, #04404483190, Roche Diagnostics, France), fasting insulin was measured by an electrochemiluminescence immunoassay (Kit Elecsys Insulin #12017547122, Roche Diagnostics, France), HbA1c was evaluated by an immunoturbidimetry and photometric assay (Kit A1C-3 #05336163190, Roche Diagnostics, France). Intra- and inter-assay coefficients of variation for fasting plasma glucose, HbA1c, and insulin measurements are provided in Supplementary Table 5. TG, total cholesterol (TC), and HDL cholesterol were measured with enzymatic colorimetric assays (Kit TRIGL #20767107322, kit CHOL2 #03039773190 and kit HDLC4 #07528566190, respectively, Roche Diagnostics, France). LDL-C was calculated with Friedewald’s formula^23^. HOMA-IR score was calculated as HOMA-IR= Insulin (mU/L) x Glucose (mg/dL) / 405^24^. Body weight (BW) and WC were measured at each visit. In a subgroup of 212 participants, an additional 2-point (before and 2 h after) oral glucose tolerance test (OGTT, 75 g) was performed at V1, V2 and V3.

### Lifestyle and dietary recommendations

Participants were dysglycemic (either with prediabetes or newly diagnosed type 2 diabetes), and did not require immediate pharmacotherapy. To ensure appropriate standard of care throughout the study, standardized lifestyle and dietary recommendations were provided to all participants. At the baseline visit, the investigator (or delegate) delivered structured counseling and provided a written handout detailing these recommendations. At each subsequent visit, the investigator (or delegate) interviewed the participant to assess adherence since the previous visit and to confirm the absence of major lifestyle changes during the preceding 72 hours. Between visits, participants received scheduled telephone follow-ups every 4 weeks to reinforce the recommendations and assess adherence. The recommendations were aligned with French public health guidance (2019) and the guidelines of the French National Health Authority (Haute Autorité de Santé, 2014). Briefly, they covered: (1) meal organization and regularity tailored to daily routines; food diversification and portion-size control; (2) food choices (reduced intake of total and saturated fats, increased intake of fiber, vegetables, and fruits; preference for unsaturated (“healthy”) fats, especially essential polyunsaturated fatty acids; choice of minimally processed foods); (3) reduction or cessation of alcohol and tobacco use; and (4) regular physical activity ( ≥ 30 min of moderate-intensity activity on ≥5 days/week, or ≥20 min of vigorous activity on ≥3 days/week), as feasible.

### Outcomes

The primary outcome was the difference in FPG change at the end of the study (V3-V1) between PBO and T63 TID. Secondary analyses included comparison with PBO of changes (V3-V1) in fasting insulin, HOMA-IR, 2-h glycemia following an OGTT, HbA1c, BW, WC, fasting TG, TC, and LDL-C, comparison with the open-label group T63 BID with PBO, and study of the effects in different subpopulations (prediabetes and T2D, based on FPG). Several secondary and exploratory outcomes deemed less relevant were not included in the present article for the sake of clarity and concision, namely: all V4 visits measurements (follow-up of a subgroup of subjects 12 weeks after the end of the study), secondary HOMA-β and QUICKI indexes, fasting free fatty acid levels, atherogenic and cardiac risk ratio indexes, fasting blood hsCRP concentration, waist to hip ratio, body composition assessed by bioelectrical impedance, and delay of occurrence of pharmacological treatment requirement for T2D. These data are available from the corresponding author upon request.

### Blood samples for fasting and post-load glucose measurements

All blood samples for fasting and post-load glucose measurements were collected under standardized conditions, identical across all study sites and in accordance with the central laboratory’s Standard Operating Procedures (SOPs). Blood was drawn into fluoride/oxalate tubes to inhibit glycolysis and centrifuged within 30 min of collection (2200 g, 15 min, 20 °C ± 2). Plasma was then aliquoted and stored at −80 °C until analysis. This procedure complies with current recommendations (1) to minimize preanalytical glucose decline due to cellular utilization.

### Nutritional intakes

Nutritional intakes were monitored quantitatively and qualitatively, on the basis of 3-day food diaries, completed in the last week before visits V1, V2 and V3 (with only one day in the week-end and at least the day just before the visit). They were analyzed by a dietician using a specialized software (NUTRILOG). Among all the data collected from these dietary survey, total energy intake, percentage of energy intake from fat, carbohydrates, and protein, intake of saturated fatty acids and intake of alcohol were statistically analyzed and used for bias factor assessment.

### Physical activity assessment

At each visit, physical activity was assessed using the International Physical Activity Questionnaire-Short Form (IPAQ-SF; 7-day recall). The investigator reviewed the questionnaire for completeness and, where necessary, clarified responses. From the IPAQ-SF, we derived the type, intensity, average duration, and frequency of the main activities (including occupational activity) performed during the week preceding each visit.

### Sample size calculation

Based on the results from a previous small pilot trial^12^, an evaluable sample of 174 participants per arm was expected to provide 88% power to detect a mean difference of 10 mg/dL in FPG value after 24 weeks between T63 TID and PBO. We assumed a standard deviation (SD) of 29.2 mg/dL in PBO and 29.7 mg/dL in T63 TID, as observed in the previous study^12^. Considering a dropout rate of 13% (12.1% in the previous study^12^), we estimated that 200 participants would be needed in each arm.

### Statistics

Continuous data are expressed as mean and standard error of mean (SEM). The baseline characteristic differences were tested with 1-way ANOVA. The analyses are presented in the intention-to-treat (ITT) population. The assumption of normality was assessed using a Shapiro-Wilk test. The comparisons between groups were performed using a mixed model. The effect of treatment was measured by time x group interaction. The investigation center was treated as random effect in addition to patient effect. Accordingly, the analysis thus accounts for clustering of participants within sites and between-site heterogeneity. The normality of residuals from random-effects models was analyzed. When appropriate, a logarithmic transformation was applied to achieve normality of the dependent variables. For the primary outcome (FPG), the results are expressed as PBO-adjusted estimated difference from mixed models (PBO-AD) with a 95% confidence interval. Sensitivity analyses were conducted using last observation carried forward (LOCF) imputation for missing data. Additional sensitivity analyses were performed with adjustment for potential confounders (total energy intake and overall physical-activity score). Finally, the primary outcome was also evaluated in the per-protocol (PP) population to corroborate the results observed in the ITT population.

In the exploratory analyses, glycemic status change was assessed in the total population classified in terms of deterioration or no deterioration (i.e. stable or improvement) of glycemic status after 6 months compared to baseline, as defined by the following FPG categories: 1/ FPG < 110 mg/dL; 2/ 110 ≤ FPG < 126 mg/dL; 3/ 126 ≤ FPG < 220 mg/dL; 4/ FPG ≥ 220 mg/dL. A Chi-squared test was used to compare glycemic status change between groups. Additionally, the relation between baseline HbA1c and HbA1c change at the end of the study was tested by the Spearman correlation coefficient.

GraphPad Prism v.9.5.0 was used to present the figures (GraphPad Software, LLC, Boston, MA, USA). The statistical analyses were performed using Stata software v.15 (StataCorp, College Station, US). The primary analysis was the comparison between the T63 TID and PBO groups. Statistical tests were two-sided with type-I error set at 5%. The widths of confidence intervals were not adjusted for multiplicity concerning secondary objectives, except for longitudinal comparisons (Sidak’s type-I error correction applied) and may not be used in place of hypothesis testing, except for the primary outcome analysis.

### Reporting summary

Further information on research design is available in the Nature Portfolio Reporting Summary linked to this article.

## Supplementary information

Supplementary Information

Reporting summary

Transparent Peer Review file

## Source data

Source Data

## Data Availability

Raw clinical data are not publicly available due to data privacy regulations. Deidentified individual participant data is available in the Source data file (all data collected during the trial). Study protocol and statistical analysis plan are also available. These data will be available immediately following publication with no end date, to anyone and for any purpose. The source data file is available indefinitely at 10.6084/m9.figshare.32348169. Source data are provided with this paper.
