# Supplementary material for: Totum-63, a plant-based polyphenol-rich extract, in prediabetes or early-stage type 2 diabetes: an international, multicenter, randomized controlled trial
Source: Nat Commun. 2026 Jul 20;17:8844. doi: 10.1038/s41467-026-75626-0 (PMC13500562; doi:10.1038/s41467-026-75626-0)
Supplement: Supplementary file 1 [file 41467_2026_75626_MOESM1_ESM.pdf]

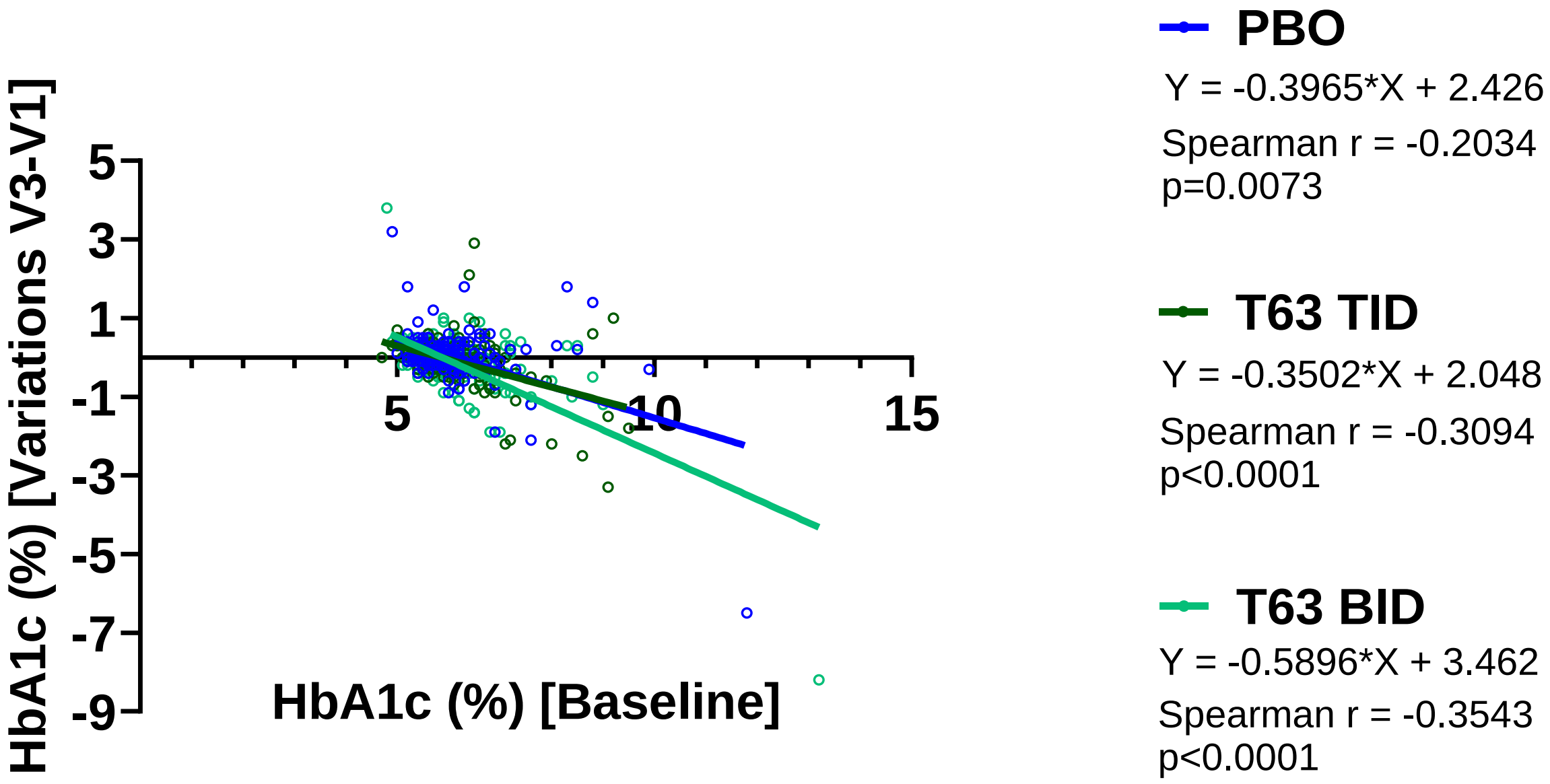

Suppl. Figure 1: Changes in HbA1c at V3 (6 months) as a function of baseline HbA1c (V1). PBO: Placebo (n=173 subjects). T63 BID: Totum-63 distributed 2 times a day (n=186 subjects). T63 TID: Totum-63 distributed 3 times a day (n=183 subjects). Strength of linear relationship (correlation coefficient, r) and significance (p) were measured by Spearman test (two sided). PBO: Placebo. T63 BID: Totum-63 distributed 2 times a day. T63 TID: Totum-63 distributed 3 times a day.

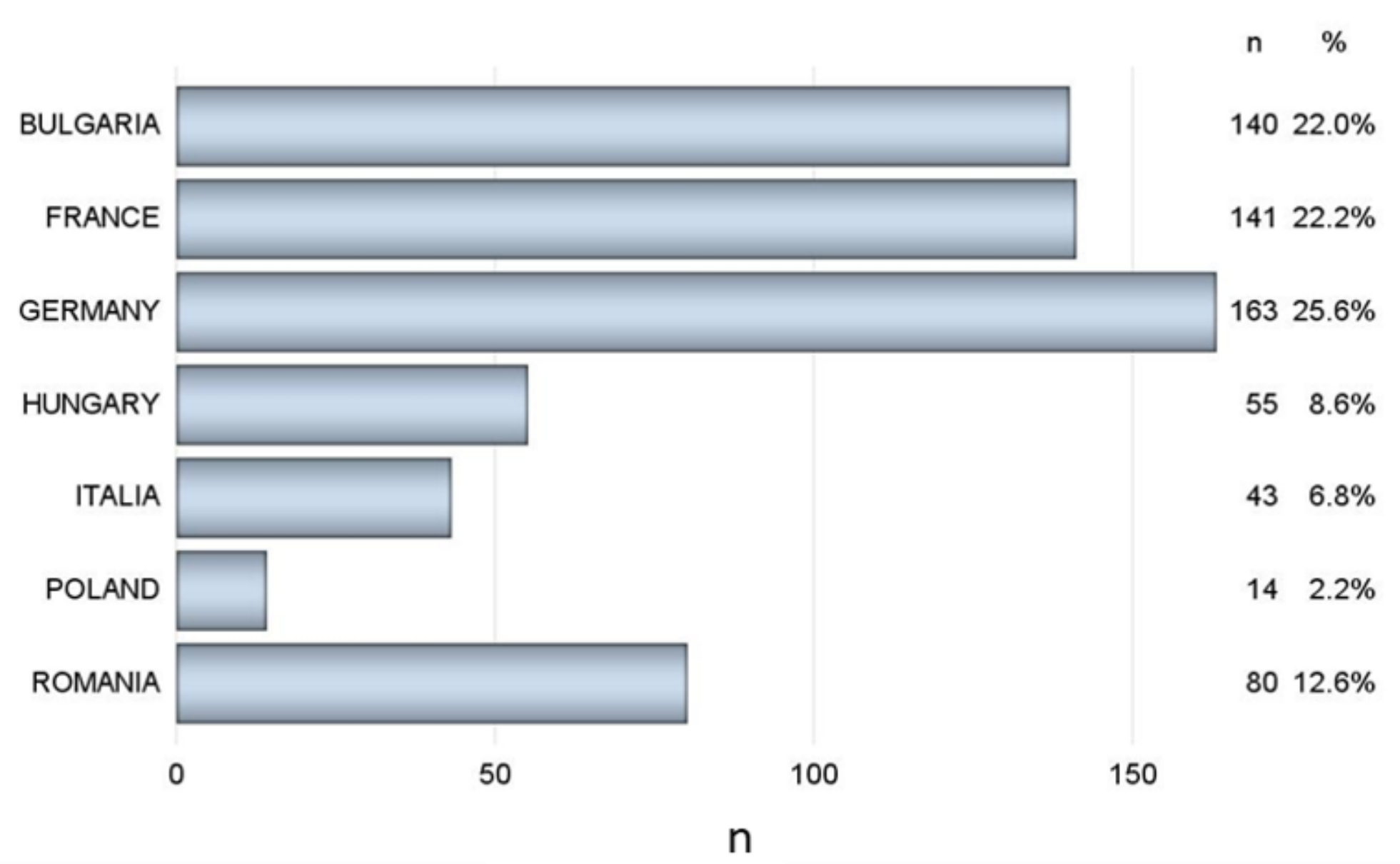

Suppl. Figure 2: Disposition of randomized subjects by country.

Supplementary table 1: Full list of inclusion and exclusion criteria.

	**Inclusion criteria**
**I1**	Age between 18 and 70 years (including ranges)
**I2**	Dysglycemic, prediabetic or newly diagnosed type 2 diabetic, without any clinical symptoms of diabetes (e.g. polyuria, polydipsia, blurred vision…) and not requiring immediate anti-diabetic treatment
**I3**	Body mass index (BMI) between 25 and 40 kg/m² (including ranges)
**I4**	Waist circumference > 102 cm for men and > 88 cm for women (-2% margin allowed, respectively ≥ 100 cm and ≥ 86.5 cm)
**I5**	Weight stable within ± 5 % in the last three months
**I6**	No significant change in food habits or in physical activity in the 3 months before the randomization and agreeing to follow hygiene and dietary (HD) recommendations given during the study
**I7**	For women: Non-menopausal with the same reliable contraception since at least three months before the beginning of the study and agreeing to keep it during the entire duration of the study (hormonal contraception, intrauterine device or surgical intervention) or menopausal with or without hormone replacement therapy (estrogenic replacement therapy begun from less than 3 months excluded)
**I8**	Good general and mental health according to the opinion of the investigator: no clinically significant and relevant abnormalities of medical history or physical examination
**I9**	Able and willing to participate to the study by complying with the protocol procedures as evidenced by his dated and signed informed consent form
**I10**	Affiliated to a social security scheme
**I11**	Agreeing to be registered on the volunteers in biomedical research (applicable only for French centers)
**I12**	Fasting plasma glucose concentration ≥ 110 mg/dL (≥ 6.10 mmol/L)

	**Exclusion criteria**
**E1**	Suffering from a metabolic disorder such as treated diabetes, uncontrolled thyroidal trouble or other metabolic disorder needing a dose adjustment in drug intervention according to the professional recommendations
**E2**	Suffering from an uncontrolled arterial hypertension (systolic blood pressure ≥ 160 mmHg and/or diastolic blood pressure ≥ 100 mmHg)
**E3**	With a history of retinopathy, ischemic cardiovascular event, having undergone recent surgical procedure in the past 6 months or in the 9 months to come
**E4**	Suffering from a severe chronic disease (e.g. cancer, HIV, renal failure, hepatic or biliary disorders ongoing, chronic inflammatory digestive disease, arthritis or other chronic respiratory trouble, etc.) or gastrointestinal disorders found to be inconsistent with the conduct of the study by the investigator (e.g. celiac disease)
**E5**	Under antidiabetic drug (e.g. biguanides, sulfonylureas, glinides, gliptines, glitazones, gliflozines, α-glucosidase inhibitors, incretins and insulin), Beta 2 agonists like salbutamol, ACE inhibitors, beta blockers, thiazide diuretics, SSIRs, MAOIs (tolerated only if stable since more than 3 months before the randomization and maintained during the whole study)
**E6**	Under lipid-lowering treatment (e.g. statins, fibrates, ezetimibe, bile acid sequestrants, niacin, etc.) since less than 3 months or modification of the treatment dose since less than 3 months before the randomization. Subject with a stable lipid-lowering treatment since at least three months can be included in the study
**E7**	Under medication which could affect glucose and/or lipid homeostasis parameters or stopped less than 3 months before randomization e.g: Neuroleptics, long-term corticosteroid systemic drugs, systemic antibodies, androgens, phenytoin, interferons, immunosuppressants, antivirals and antiretrovirals, are not allowed during the study
**E8**	Regular intake of dietary supplements or "health foods" or products rich in plant stanol or sterol (like PRO-ACTIV® or DANACOL® products), rich in long chain omega-3 fatty acids (especially soft gels containing fish oils), or in other substances intended to reduce glycemia (e.g. beta-glucans, konjac, olive leaf extract, berberine, cinnamon, etc.) or stopped less than 3 months before the randomization

**E9**	Under treatment or dietary supplement which could significantly affect parameter(s) followed during the study according to the investigator or stopped in a too short period before the randomization (for example consumed in the month before randomization)
**E10**	With a known or suspected food allergy or intolerance or hypersensitivity to any of the study products' ingredient
**E11**	Consuming more than 3 standard drinks daily of alcoholic beverage for men or 2 standard drinks daily for women
**E12**	With extreme and/or unbalanced eating habits (vegetarian, vegan, skipping meals regularly)
**E13**	With a personal history of anorexia nervosa, bulimia or significant eating disorders according to the investigator
**E14**	Smoking more than 20 cigarettes daily. The subject should be able not to smoke during the visits (maximum 4 hours)
**E15**	Having a lifestyle deemed incompatible with the study according to the investigator including high level of physical activity (defined as more than 10 hours of intense physical activity a week, walking excluded)
**E16**	Pregnant (as evidenced by a positive test for β-HCG, i.e. > 5 mUI/mL, realized at V0) or lactating women or intending to become pregnant within 9 months ahead
**E17**	Who made a blood donation in the 3 months before the randomization or intending to make it within 9 months ahead
**E18**	Taking part in another clinical trial or being in the exclusion period of a previous clinical trial
**E19**	Having received, during the last 12 months, indemnities for clinical trial higher or equal to 4500 Euros (applicable only for French centers)
**E20**	Under legal protection (guardianship, wardship) or deprived from his rights following administrative or judicial decision
**E21**	Presenting a psychological or linguistic incapability to sign the informed consent
**E22**	Impossible to contact in case of emergency
**E23**	Any condition assessed by the investigator which could endanger patient's safety or the conduct of the study (e.g. device related contraindication for impedancemetry and/or DEXA such as pacemaker or electronic implant)
	**At V0**
**E24**	Fasting glucose plasma concentration > 220 mg/dL (> 12.2 mmol/L)
**E25**	Fasting blood triglycerides > 2.2 g/L (> 2.48 mmol/L)
**E26**	TSH outside the laboratory normal values
**E27**	Fasting blood LDL cholesterol > 1.9 g/L (> 4.88 mmol/L) or non HDLc > 2.2 g/L (> 5.65 mmol/L) or any condition requiring a therapeutic dose adjustment during the trial according to the professional recommendations
**E28**	Blood AST, ALT or GGT > 3xULN (Upper Limit of Normal)
**E29**	Blood creatinine concentration > 125 µmol/L
**E30**	eGFR (calculated by CKD-EPI formula) < 60 mL/min/1.73m²
**E31**	Complete blood count (CBC) with hemoglobin < 11 g/dL (< 6.83 mmol/L) or leucocytes < 3000 /mm3 (< 3 G/L; < 3 /µL) or leucocytes > 16000 /mm3 (> 16 G/L; > 16 /µL) or clinically significant abnormality according to the investigator

Supplementary table 2: Secondary analyses, change at 3 months. Table 2: Values are presented as mean (SEM). Comparisons were made using a linear mixed model on log-transformed values considering baseline (V1), 3 months (V2) and 6-months (V3) values. The number of subjects, denoted as "n," is presented as an interval representing the minimum and maximum number of participants for each parameter, accounting for variations across visits (V1 to V3) due to missing values. Intergroup comparisons vs. PBO presented in the table were performed on the baseline-adjusted 3-month difference (V2-V1 change), and significant p-values (<0.05) are highlighted in bold characters. Intragroup comparisons versus baseline (within-group changes) were obtained as 6-month-versus-baseline contrasts from the same mixed model; they are indicated by asterisks are indicated by asterisks (*p<0.05, **p<0.01, ***p<0.001, ****p<0.0001). Statistical tests were two-sided with type-I error set at 5%. Fasting ins.: fasting insulin. PBO: Placebo. T63 BID: Totum-63 distributed 2 times a day. T63 TID: Totum-63 distributed 3 times a day. WC: waist circumference. 2-h gly.: 2-hour glycemia following oral glucose tolerance test. Source data are provided as a Source Data file.

		PBO			T63 TID			T63 BID		
		Total population	Prediabetes	Type 2 diabetes	Total population	Prediabetes	Type 2 diabetes	Total population	Prediabetes	Type 2 diabetes
**FPG (mg/dL)**		n=198-209	n=153-161	n=45-48	n=202-212	n=164-172	n=38-40	n=203-214	n=158-167	n=45-47
Baseline	Mean (SEM)	122.0 (1.6)	115.2 (1.1)	145.1 (4.5)	122.0 (1.5)	115.8 (1.1)	148.4 (4.6)	124.4 (1.5)	118.6 (1.1)	145.0 (4.3)
3 months	Mean (SEM)	122.2 (1.7)	117.2 (1.5)	138.9 (4.3)	120.1 (1.8)	114.2 (1.4)	146.0 (5.8)	121.7 (1.8)*	115.3 (1.1)**	144.2 (5.8)
P-value (change 3 months-baseline, vs placebo, raw)					*0.1537*	***0.0402***	*0.7299*	***0.0473***	***0.0061***	*0.7062*
**Fasting ins. (mU/L)**		n=197-208	n=152-160	n=45-48	n=203-212	n=165-172	n=37-40	n=203-214	n=158-167	n=45-47
Baseline	Mean (SEM)	18.30 (0.87)	17.47 (0.87)	21.07 (2.39)	18.16 (0.79)	17.81 (0.86)	19.67 (2.00)	18.88 (0.94)	18.59 (1.02)	19.90 (2.28)
3 months	Mean (SEM)	18.30 (0.91)	17.83 (1.09)	19.86 (1.50)	17.37 (0.78)	16.77 (0.77)	20.01 (2.50)	17.32 (0.84)**	16.72 (0.96)**	19.41 (1.73)
P-value (change 3 months-baseline, vs placebo, raw)					*0.5271*	*0.4941*	*0.9984*	*0.1162*	*0.0612*	*0.8521*
**HOMA-IR**		n=197-208	n=152-160	n=45-48	n=202-212	n=164-172	n=37-40	n=202-214	n=157-167	n=45-47
Baseline	Mean (SEM)	5.60 (0.28)	5.02 (0.26)	7.53 (0.81)	5.56 (0.26)	5.19 (0.27)	7.13 (0.73)	5.83 (0.30)	5.47 (0.30)	7.09 (0.82)
3 months	Mean (SEM)	5.76 (0.39)	5.38 (0.46)	7.04 (0.66)	5.37 (0.31)	4.87 (0.27)	7.51 (1.09)	5.31 (0.29)**	4.85 (0.31)***	6.91 (0.70)
P-value (change 3 months-baseline, vs placebo, raw)					*0.3585*	*0.2737*	*0.8979*	***0.0477***	***0.0163***	*0.7561*
**2-h gly. (mg/dL)**		n=65-70	n=54-58	n=11-12	n=68-69	n=58-59	n=10	n=68-73	n=55-60	n=13
Baseline	Mean (SEM)	149.1 (7.9)	138.7 (7.1)	199.5 (27.8)	154.2 (6.6)	146.9 (6.8)	196.9 (17.4)	155.7 (6.4)	151.2 (6.5)	176.3 (19.7)
3 months	Mean (SEM)	150.0 (8.0)	137.3 (6.3)	210.4 (30.7)	148.6 (7.8)	135.2 (5.7)	226.9 (33.2)	139.2 (5.7)*	135.3 (5.5)	156.1 (18.8)
P-value (change 3 months-baseline, vs placebo, raw)					*0.4048*	*0.3143*	*0.8164*	*0.1196*	*0.2267*	*0.2536*

		PBO			T63 TID			T63 BID		
		Total population	Prediabetes	Type 2 diabetes	Total population	Prediabetes	Type 2 diabetes	Total population	Prediabetes	Type 2 diabetes
**HbA1c (%)**		n=173-186	n=136-145	n=37-41	n=183-194	n=147-156	n=35-38	n=186-197	n=145-154	n=41-43
Baseline	Mean (SEM)	6.10 (0.06)	5.87 (0.04)	6.90 (0.21)	6.09 (0.06)	5.90 (0.05)	6.90 (0.18)	6.14 (0.06)	5.93 (0.04)	6.86 (0.20)
3 months	Mean (SEM)	6.05 (0.05)	5.90 (0.04)	6.57 (0.17)	6.00 (0.05)	5.85 (0.04)	6.66 (0.15)	6.06 (0.05)	5.86 (0.04)	6.76 (0.16)
	P-value (change 3 months-baseline, vs placebo, raw)				*0.4327*	*0.2117*	*0.9571*	*0.3512*	*0.1529*	*0.9403*
**Body weight (kg)**		n=198-210	n=153-162	n=45-48	n=205-212	n=167-172	n=38-40	n=204-214	n=159-167	n=45-47
Baseline	Mean (SEM)	93.04 (1.10)	92.96 (1.26)	93.29 (2.22)	93.55 (1.18)	92.67 (1.28)	97.30 (2.92)	90.36 (1.10)	89.33 (1.20)	94.03 (2.57)
3 months	Mean (SEM)	92.40 (1.14)**	92.56 (1.31)*	91.86 (2.28)	93.10 (1.19)**	92.25 (1.28)*	96.81 (3.06)**	89.48 (1.12)***	88.47 (1.21)***	93.11 (2.68)
	P-value (change 3 months-baseline, vs placebo, raw)				*0.7621*	*0.9569*	*0.2914*	*0.4912*	*0.4008*	*0.8919*
**WC (cm)**		n=198-210	n=153-162	n=45-48	n=204-212	n=166-172	n=38-40	n=203-214	n=158-167	n=45-47
Baseline	Mean (SEM)	109.4 (0.9)	109.2 (1.0)	110.2 (1.6)	108.7 (0.9)	107.6 (0.9)	113.5 (2.4)	107.4 (0.8)	106.8 (0.9)	109.7 (1.8)
3 months	Mean (SEM)	108.2 (0.9)****	108.2 (1.1)***	108.5 (1.8)**	108.0 (0.9)***	106.8 (0.9)***	113.3 (2.5)	106.6 (0.8)***	105.8 (0.9)**	109.4 (1.8)
	P-value (change 3 months-baseline, vs placebo, raw)				*0.5950*	*0.9527*	*0.3349*	*0.3880*	*0.7743*	*0.2166*
**TG (g/L)**		n=198-209	n=153-161	n=45-48	n=204-212	n=166-172	n=38-40	n=202-212	n=158-166	n=44-46
Baseline	Mean (SEM)	1.35 (0.06)	1.33 (0.07)	1.42 (0.11)	1.33 (0.05)	1.28 (0.06)	1.52 (0.10)	1.28 (0.04)	1.25 (0.04)	1.40 (0.11)
3 months	Mean (SEM)	1.33 (0.06)	1.30 (0.07)	1.43 (0.10)	1.23 (0.05)**	1.20 (0.05)*	1.37 (0.12)	1.26 (0.05)	1.22 (0.06)	1.43 (0.11)
	P-value (change 3 months-baseline, vs placebo, raw)				*0.0847*	*0.3463*	*0.0566*	*0.5957*	*0.7302*	*0.6622*
**TC (g/L)**		n=198-209	n=153-161	n=45-48	n=204-212	n=166-172	n=38-40	n=203-213	n=159-167	n=44-46
Baseline	Mean (SEM)	1.90 (0.03)	1.91 (0.03)	1.88 (0.06)	1.92 (0.03)	1.92 (0.03)	1.93 (0.08)	1.92 (0.03)	1.93 (0.03)	1.87 (0.06)
3 months	Mean (SEM)	1.93 (0.03)	1.96 (0.03)	1.85 (0.06)	1.83 (0.03)**	1.84 (0.03)*	1.78 (0.07)	1.92 (0.03)	1.92 (0.03)	1.91 (0.07)
	P-value (change 3 months-baseline, vs placebo, raw)				***0.0017***	***0.0037***	*0.1668*	*0.2787*	*0.1169*	*0.5751*
**LDL-C (g/L)**		n=191-204	n=148-158	n=43-46	n=199-210	n=161-170	n=37-40	n=198-210	n=155-165	n=43-45
Baseline	Mean (SEM)	1.15 (0.02)	1.15 (0.03)	1.14 (0.05)	1.16 (0.03)	1.16 (0.03)	1.14 (0.06)	1.17 (0.03)	1.19 (0.03)	1.10 (0.06)
3 months	Mean (SEM)	1.17 (0.02)	1.18 (0.03)	1.10 (0.05)	1.09 (0.02)**	1.10 (0.03)**	1.04 (0.06)	1.16 (0.03)	1.16 (0.03)	1.15 (0.05)
	P-value (change 3 months-baseline, vs placebo, raw)				***0.0065***	***0.0055***	*0.4985*	*0.4404*	*0.0900*	*0.1527*

Supplementary table 3: Changes in total energy intake (dietary questionnaires) and total metabolic equivalents (physical activity questionnaire). Values are presented as means (SEM). Comparisons were made with a linear mixed model on log-transformed values considering baseline (V1), 3 months (V2) and 6-months (V3) values. The number of subjects (n) is reported as a range (min–max) for each parameter to account for visit-to-visit variations (V1–V3) due to missing data. IPAQ: International Physical Activity Questionnaire. PBO: Placebo. T63 BID: Totum-63 distributed 2 times a day. T63 TID: Totum-63 distributed 3 times a day. Source data are provided as a Source Data file.

		PBO	T63 TID	T63 BID
**Total energy intake (kcal/day)**		n=179-202	n=189-199	n=188-204
Baseline	Mean (SEM)	1669±44	1718±42	1685±42
3 months	Mean (SEM)	1621±51	1666±41	1584±42
6 months	Mean (SEM)	1620±46	1616±39	1572±41
P-value (Mixed Model Group/Time interaction vs placebo)			*0.492*	*0.406*
**IPAQ score (MET-min/week)**		n=167-188	n=184-185	n=178-184
Baseline	Mean (SEM)	3965±368	3879±329	4029±398
3 months	Mean (SEM)	4454±374	4183±369	4142±342
6 months	Mean (SEM)	4012±359	4147±351	3577±358
P-value (Mixed Model Group/Time interaction vs placebo)			*0.753*	*0.418*

Supplementary table 4: Descriptive statistics of treatment-emergent adverse events (TEAEs) by body system (In the safety population). PBO: Placebo. T63 BID: Totum-63 distributed 2 times a day. T63 TID: Totum-63 distributed 3 times a day.

	**PBO (n=210)**		**T63 BID (n=214)**		**T63 TID (n=212)**	
	**Subjects n (%)**	**Events n**	**Subjects n (%)**	**Events n**	**Subjects n (%)**	**Events n**
**All treatment-emergent adverse events**	70 (33.3%)	179	77 (36.0%)	188	82 (38.7%)	175
**System**						
Allergy	2 (1.0%)	2	1 (0.5%)	1	3 (1.4%)	3
Cardiovascular	4 (1.9%)	4	2 (0.9%)	2	2 (0.9%)	3
Dermatological	5 (2.4%)	5	7 (3.3%)	10	7 (3.3%)	7
Endocrine / Hematologic	2 (1.0%)	2	1 (0.5%)	1	2 (0.9%)	2
ENT / Ocular	9 (4.3%)	9	15 (7.0%)	19	5 (2.4%)	6
Gastro-intestinal	23 (11.0%)	39	27 (12.6%)	35	30 (14.2%)	41
Gynecological	2 (1.0%)	2	3 (1.4%)	3	1 (0.5%)	2
Immunological	6 (2.9%)	7	11 (5.1%)	14	10 (4.7%)	14
Locomotor / Rheumatologic	18 (8.6%)	32	27 (12.6%)	44	22 (10.4%)	31
Nephro-urological	5 (2.4%)	5	3 (1.4%)	3	3 (1.4%)	4
Neurologic / Psychiatric	18 (8.6%)	36	14 (6.5%)	26	14 (6.6%)	31
Metabolic	4 (1.9%)	5	1 (0.5%)	1	1 (0.5%)	1
Respiratory	13 (6.2%)	13	20 (9.3%)	21	16 (7.5%)	18
Other	13 (6.2%)	18	6 (2.8%)	8	11 (5.2%)	12

Supplementary table 5: Intra- and inter-assay coefficients of variation for fasting plasma glucose, HbA1c, and insulin measurements

Analyte	Instrument / Method	Control level	Intra-assay CV (%)	Inter-assay CV (%)
**Fasting plasma glucose**	Cobas Integra 400+, hexokinase (Roche Diagnostics)	5.56 mmol/L	1.08	2.7
		13.46 mmol/L	0.26	2.6
**HbA1c**	A1C-3 immunoturbidimetric assay (Roche Diagnostics)	5.59%	0.83	0.62
		10.67%	1.24	1.94
**Insulin**	Elecsys electrochemiluminescent immunoassay (Roche Diagnostics)	19.6 µIU/mL	1.19	0.8
		96 µIU/mL	1.54	1.0

Supplementary table 6: Detailed components of the physical activity score

Values are presented as means (SEM). Comparisons were made with a linear mixed model on log-transformed values considering baseline (V1), 3 months (V2) and 6-months (V3) values. The number of subjects (n) is reported as a range (min–max) for each parameter to account for visit-to-visit variations (V1–V3) due to missing data. MET: Metabolic Equivalent of Task. PBO: Placebo. T63 BID: Totum-63 distributed 2 times a day. T63 TID: Totum-63 distributed 3 times a day. Source data are provided as a Source Data file.

		PBO	T63 TID	T63 BID
**Score of vigorous intensity (MET-minutes/week)**		n=188-203	n=198-206	n=190-202
Baseline	Mean (SEM)	993±192	1052±207	1018±217
3 months	Mean (SEM)	1260±205	1044±203	908±136
6 months	Mean (SEM)	936±172	1030±175	922±199
P-value (Mixed Model Group/Time interaction vs placebo)			*0.818*	*0.419*
**Score of moderate intensity (MET-minutes/week)**		n=183-200	n=194-199	n=191-199
Baseline	Mean (SEM)	1216±145	1162±119	1218±146
3 months	Mean (SEM)	1405±143	1375±154	1506±168
6 months	Mean (SEM)	1316±164	1441±165	1084±132
P-value (Mixed Model Group/Time interaction vs placebo)			*0.876*	*0.300*
**Score of walking (MET-minutes/week)**		n=175-191	n=186-188	n=183-189
Baseline	Mean (SEM)	1597±132	1551±130	1690±187
3 months	Mean (SEM)	1650±134	1617±152	1585±126
6 months	Mean (SEM)	1701±155	1565±136	1489±138
P-value (Mixed Model Group/Time interaction vs placebo)			*0.699*	*0.817*

Supplementary table 7: Detailed components of the dietary variables

Values are presented as means (SEM). Comparisons were made with a linear mixed model on log-transformed values considering baseline (V1), 3 months (V2) and 6-months (V3) values. The number of subjects (n) is reported as a range (min–max) for each parameter to account for visit-to-visit variations (V1–V3) due to missing data. PBO: Placebo. T63 BID: Totum-63 distributed 2 times a day. T63 TID: Totum-63 distributed 3 times a day. Source data are provided as a Source Data file.

		PBO	T63 TID	T63 BID
**Carbohydrates (g/day)**		n=179-202	n=189-199	n=188-204
Baseline	Mean (SEM)	166±5	171±5	171±5
3 months	Mean (SEM)	159±6	160±5	159±5
6 months	Mean (SEM)	161±6	158±5	163±5
P-value (Mixed Model Group/Time interaction vs placebo)			*0.230*	*0.528*
**Proteins (g/day)**		n=179-202	n=189-199	n=188-204
Baseline	Mean (SEM)	78±2	78±2	76±2
3 months	Mean (SEM)	76±2	80±2	73±2
6 months	Mean (SEM)	78±2	76±2	73±2
P-value (Mixed Model Group/Time interaction vs placebo)			*0.117*	*0.670*
**Fat (g/day)**		n=179-202	n=189-199	n=188-204
Baseline	Mean (SEM)	68±2	71±2	68±2
3 months	Mean (SEM)	68±2	70±2	65±2
6 months	Mean (SEM)	66±2	66±2	62±2
P-value (Mixed Model Group/Time interaction vs placebo)			*0.415*	*0.503*
**Alcohol (g/day)**		n=179-202	n=189-199	n=188-204
Baseline	Mean (SEM)	4.1±0.6	5.2±0.8	5.1±0.8
3 months	Mean (SEM)	4.0±0.6	5.3±0.7	4.8±0.8
6 months	Mean (SEM)	4.7±0.6	6.5±0.9	3.6±0.5
P-value (Mixed Model Group/Time interaction vs placebo)			*0.766*	*0.475*
**Fiber (g/day)**		n=179-202	n=189-199	n=188-204
Baseline	Mean (SEM)	18.8±0.6	18.7±0.6	18.5±0.6
3 months	Mean (SEM)	17.6±0.6	17.8±0.6	17.5±0.6
6 months	Mean (SEM)	17.5±0.6	17.7±0.6	17.4±0.6
P-value (Mixed Model Group/Time interaction vs placebo)			*0.295*	*0.927*
**Saturated fatty acids (g/day)**		n=179-202	n=189-199	n=188-204
Baseline	Mean (SEM)	27.0±0.9	28.5±1.0	28.0±1.0
3 months	Mean (SEM)	26.7±1.1	28.7±1.1	26.1±0.9
6 months	Mean (SEM)	25.6±0.8	26.7±0.9	25.8±0.9
P-value (Mixed Model Group/Time interaction vs placebo)			*0.328*	*0.622*

Reference:

1. Sacks et al. (2023): Guidelines and Recommendations for Laboratory Analysis in the Diagnosis and Management of Diabetes Mellitus. Clinical Chemistry 69:8 p.808–868.
