# Supplementary material for: Totum-63, a plant-based polyphenol-rich extract, in prediabetes or early-stage type 2 diabetes: an international, multicenter, randomized controlled trial
Source: Nat Commun. 2026 Jul 20;17:8844. doi: 10.1038/s41467-026-75626-0 (PMC13500562; doi:10.1038/s41467-026-75626-0)
Supplement: Supplementary file 3 [file 41467_2026_75626_MOESM3_ESM.pdf]

nature portfolio

Peer Review File

Totum-63, a plant-based polyphenol-rich extract, in prediabetes or early-stage type 2 diabetes: an international, multicenter, randomized controlled trial

Corresponding Author: Dr Andre Marette

This file contains all reviewer reports in order by version, followed by all author rebuttals in order by version.

Version 0:

Reviewer comments:

Reviewer #1

(Remarks to the Author)
Thank you for this well-executed and clearly written manuscript. The study addresses an important gap in non-pharmacological strategies for early dysglycemia and contributes valuable multicenter RCT data. I appreciate the comprehensive reporting and the rigor of the primary outcome analysis. I have two suggestions and questions for clarification that I hope may strengthen the presentation of your findings:

Site-level recruitment and adjustment (Lines ~80–119):
Would it be possible to provide more detail about how many participants were recruited per site or per country? This may help the reader assess geographic distribution and generalizability. In addition, was any adjustment performed for site effect in the primary or secondary analyses? This could be particularly relevant given the multicenter design.

Potential consideration for multiple testing (Lines ~174–177):
Th authors stated the widths of confidence intervals were not adjusted for multiplicity concerning secondary objectives, except for longitudinal comparisons. What was the reason behind it? Given the number of secondary outcomes assessed, did the authors attempt applying a formal correction for multiple comparisons? If not, a brief justification or sensitivity analysis could reassure readers that the secondary findings are robust.

Reviewer #3

(Remarks to the Author)
To the authors,

The randomized controlled trial, “Effects of Totum-63 on fasting plasma glucose in individuals with dysglycemia: a large-scale randomized double-blind placebo-controlled trial.”, conducted by Chavanelle et al. investigated the efficacy of Totum-63 (T63) in improving FPG in subjects with prediabetes or early-stage T2D. After six months, FPG was significantly reduced in both the T63 TID and BID groups compared to placebo. T63 also improved HbA1c, glucose tolerance, body weight, and lipid profile, with the extent of these benefits varying by dosing schedule (BID vs. TID). Although the novel compound is of interest, I may share the following concerns:

- The authors list “no significant change in food habits or physical activity in the 3 months prior” as an inclusion criterion; however, lifestyle adjustments should have been actively implemented and standardized before trial initiation, as is common in trials of glucose-lowering agents. This helps ensure a uniform baseline and allows the drug’s effect to be isolated. Instead, the additional provision of “general lifestyle recommendations” introduces uncontrolled variability from baseline, potentially confounding the observed outcomes.

- Although the study showed statistical significance in its primary outcome, the absolute effect size was relatively small (1.9 and 4.25 mg/dL in the TID and BID groups, respectively) and notably lower than the expected target of 10 mg/dL. The sample size was calculated to provide 88% power to detect a 10 mg/dL difference in FPG between T63 TID and placebo at

24 weeks. However, the observed treatment effect was substantially smaller than anticipated. As a result, the study was likely overpowered, and the statistical significance of the findings should be interpreted with caution, as even clinically marginal differences may reach significance in such settings.

- The authors state that their results are comparable to those of the meta-analysis by Zhao et al. However, this comparison is only applicable to prediabetic patients, in whom exercise and metformin were associated with FPG redctions of −0.26 mmol/L (about -4.7 mg/dL) and −0.33 mmol/L (about -5.9 mg/dL), respectively. Hence, the treatment effect observed in the exercise alone group was greater than that observed in the present study. Importantly, in the study of Zhao et al., patients with T2D, patients had a reduction of FPG of −33.84 mg/dL and −33.30 mg/dL in the exercise and metformin groups, respectively, thus greatly surpassing the treatment effect noted in the present study.

- Among 1,846 subjects screened, only 636 were eligible and randomized. Given that the drug appears to target a broad population, this high screen failure rate raises concerns about the representativeness of the enrolled cohort. Such selective inclusion may limit the external validity and generalizability of the findings—particularly to real-world patients with comorbidities or diabetes-related complications.

Minor Comments:
- Please provide a rationale for why the BID regimen group was open-label

- Describe if T63 is expected to have any drug-drug interactions with other substances

- Consider providing a more informative table description for Table 2

- The formatting of Table 4 is unclear, as the asterisk symbols (e.g., *) do not consistently correspond to the p-values listed below. Please clarify their meaning in the table legend.

Additional comment
- The press release information found under https://investisseurs.valbiotis.com/en/totum-63/ references this study and published claims not justified by the present data. For example, it states that T63 significantly reduces fasting blood glucose (-8.1 mg/dl), 2-hour blood glucose
(-21.9 mg/dl), glycated hemoglobin (-0.18%) and the HOMA-IR insulin resistance score (-1.04 pts), compared with placebo. group and placebo. The actual reduction from baseline within the T63 group is substantially smaller, and should be used for a proper interpretation of the treatment effect. Furthermore, the authors claim a significantly reduced progression towards type 2 diabetes, an outcome not investigated in the present study (only deterioration in glycemic status was assessed). A final example is found in the claim that “The study confirms TOTUM63’s excellent safety profile, with no risk of hypoglycemia..”. Again, the present study did not investigate the association between T63 and risk of hypoglycemia. Finally, the product is labeled as a dietary supplement, in which it is strictly prohibited to claim disease prevention or treatment. Such claims are reserved exclusively for drugs that have undergone rigorous FDA review and approval.

Reviewer #4

(Remarks to the Author)
I co-reviewed this manuscript with one of the reviewers who provided the listed reports. This is part of the Nature Communications initiative to facilitate training in peer review and to provide appropriate recognition for Early Career Researchers who co-review manuscripts.

Reviewer #5

(Remarks to the Author)
Effects of Totum-63 on fasting plasma glucose in individuals with dysglycemia: a large- scale randomized double-blind placebo-controlled trial by V Chavanelle et al.

A multicentre RCT of a plant-based product (3-times a day) was conducted with 6 months intervention in adults with pre-diabetes or Type 2 diabetes was conducted. Individuals from 37 seven European countries with FPG between 110-220 mg/dL were randomized to receive 38 placebo (PBO, n=210), T63 (5 g/d) distributed thrice daily (T63 TID, n=212) or twice daily 39 (T63 BID, open-label arm, n=214) over six months. The primary outcome was the FPG change in T63 TID compared to PBO in the intention-to-treat population. Secondary assessments included changes in diabetes biomarkers, lipid profile, anthropometric measurements and subpopulation analyses based on glycemia. After 6-months, FPG was significantly reduced in the T63 TID group (PBO-adjusted mixed model estimated difference, PBO-AD=-44 3.8 mg/dL, 95% CI: [-5.9; -1.7], p=0.0149) and the T63 BID group (PBO-AD=-5.5 mg/dL, 45 95% CI: [-9.7; -1.3] p<0.001). T63 also improved HbA1c, glucose tolerance, body weight and lipids, with magnitude of benefits differing based on the distribution pattern. Subpopulation analyses showed efficacy of T63 in subjects with prediabetes.

Comments: Pre-diabetes and Type 2 diabetes are pandemic and a major cause of morbidity, premature mortality and high socioeconomic costs. Treatments to prevent progression of dysglycaemia are needed.

There was no run-in period with lifestyle changes only, and this and the Hawthorne effect could contribute to the benefits

noted, even though there is a placebo group.

Plant extracts often have an odour or taste, and some may change urine colour. Was the active drug and placebo identical in smell, taste and appearance and was there any urine or faeces colour change with the active drugs.

What was the time between blood being drawn and separated, as beyond a certain time the plasma or serum glucose level drops due to cellular utilisation?

Does T63 affect any of the assays in particular, glucose, HbA1c and insulin? Tests such as assays without and with the addition of T63 to blood are of interest.

Was the HbA1c assay DCCT aligned?

What were the intra and inter-assay CVs for each of the assays, and were all samples from a participant on the same run?

Continuous Glucose Monitoring (CGM) metrics would have been very valuable in the glucose profiling of the study subjects. Why was CGM not used to assess glycaemia?

The adverse events are described by severity, not by what they were. Please do so. What type of events were the ones that continued till trial end?

Version 1:

Reviewer comments:

Reviewer #1

(Remarks to the Author)
I am generally satisfied with the answers to my comments and the revision from the research team.

Reviewer #3

(Remarks to the Author)
The authors did their best to address my concerns in their cover letter, but changed little in the paper. All major concerns still remain to me.

Lifestyle-confounding concerns: the reply by the authors is long, but it does not resolve several key issues, e.g. they rely almost entirely on self-reported diet and physical activity. RCT internal validity takes precedence over mirroring real-world practice when mechanistic isolation of treatment effects is the goal.

Defense of the small effect size: they sidestep the issue that statistical ≠ clinical significance, and their reply does not provide a robust framework for interpreting the modest effect.

In Zhao et al. treatment effect observed in the exercise alone group was greater than that observed in the present study: they spend most of the response constructing post-hoc adjusted comparisons using Zhao's regression coefficient to estimate what exercise/metformin "would" do at this study's baseline FPG.

A 65% screening failure rate still raises concerns about generalizability, although I can accept the reason for the main source of screening failure.

Reviewer #5

(Remarks to the Author)
I have reviewed the authors' responses and edited manuscript, including the much expanded supplementary materials. My queries have been adequately addressed and I have no concerns to raise regarding the other aspects of the revised manuscript.

Reviewer #6

(Remarks to the Author)
The authors responded effectively to Reviewer 3. They addressed the concerns directly, used their own data where possible, and strengthened their argument by referencing established literature. The revisions also improve the clarity of the paper and make the conclusions easier to justify.

Open Access This Peer Review File is licensed under a Creative Commons Attribution 4.0 International License, which permits use, sharing, adaptation, distribution and reproduction in any medium or format, as long as you give appropriate credit to the original author(s) and the source, provide a link to the Creative Commons license, and indicate if changes were made.
In cases where reviewers are anonymous, credit should be given to 'Anonymous Referee' and the source.
The images or other third party material in this Peer Review File are included in the article's Creative Commons license, unless indicated otherwise in a credit line to the material. If material is not included in the article's Creative Commons license and your intended use is not permitted by statutory regulation or exceeds the permitted use, you will need to obtain permission directly from the copyright holder.
To view a copy of this license, visit https://creativecommons.org/licenses/by/4.0/

Reviewers' comments:

NB: All changes in the manuscript are highlighted in yellow.

Reviewer #1 (Remarks to the Author):

Thank you for this well-executed and clearly written manuscript. The study addresses an important gap in non-pharmacological strategies for early dysglycemia and contributes valuable multicenter RCT data. I appreciate the comprehensive reporting and the rigor of the primary outcome analysis.

Thank you very much for reviewing our manuscript and for your constructive comments and suggestions.

I have two suggestions and questions for clarification that I hope may strengthen the presentation of your findings:

Site-level recruitment and adjustment (Lines ~80–119):

Would it be possible to provide more detail about how many participants were recruited per site or per country? This may help the reader assess geographic distribution and generalizability.

We agree that this information may be of interest to readers. The figure below shows the distribution of subjects by country. Among the seven countries represented, the three main contributors are Germany (n=163), France (n=141), and Bulgaria (n=140). Excluding Poland, which contributed only marginally (n=14), **the other six countries contributed substantially, thereby providing reasonably good representativeness of the European population**. This figure has been added to the Supplementary Materials (Page 4, Supplementary Figure 2) to make it available to readers.

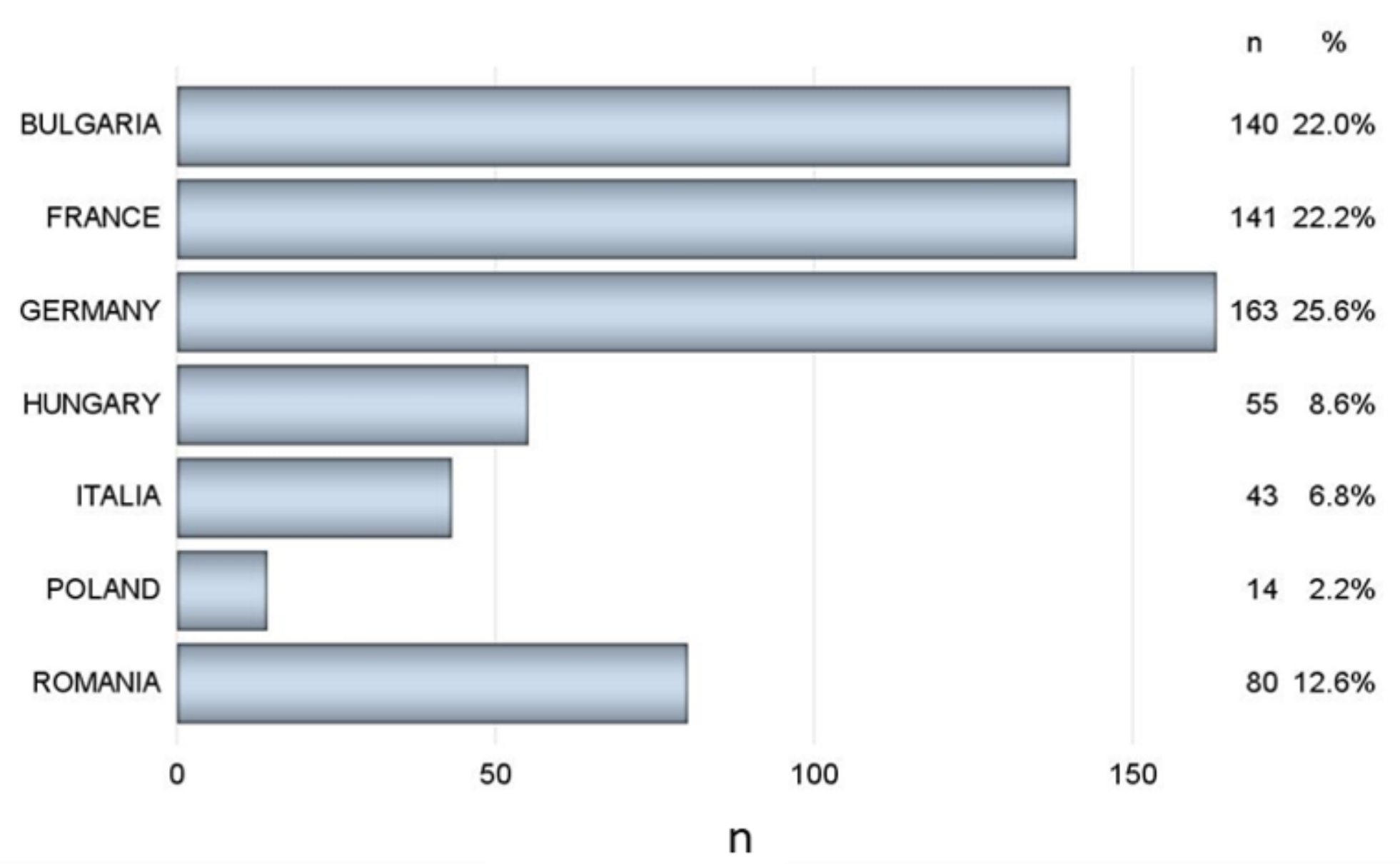

In addition, was any adjustment performed for site effect in the primary or secondary analyses? This could be particularly relevant given the multicenter design.

We thank the reviewer for this comment. As detailed in the manuscript, **all primary and secondary analyses were conducted using mixed-effects models with study site specified as a random intercept, thereby accounting for clustering of participants within sites and for between-site heterogeneity (i.e., both within- and between-site variability)**. We have added a clarifying sentence to the Statistical Analysis section of the manuscript (Methods; Page 6, line 166 in the revised version) to clarify this point.

Potential consideration for multiple testing (Lines ~174–177):

The authors stated the widths of confidence intervals were not adjusted for multiplicity concerning secondary objectives, except for longitudinal comparisons. What was the reason behind it? Given the number of secondary outcomes assessed, did the authors attempt applying a formal correction for multiple comparisons? If not, a brief justification or sensitivity analysis could reassure readers that the secondary findings are robust.

We thank the reviewer for this comment. Statistical analysis plan and the realization of **statistical analysis have been conducted by an independent academic center** (Biostatistical unit of the University Hospital of Clermont-Ferrand, directed by Dr. Bruno Pereira, coordinating biostatistician). Bruno Pereira's team has published these last years many randomized clinical trial using this statistical methodology (no adjustment for multiplicity for secondary outcomes), for example:

- N Engl J Med. 2023 Nov 9;389(19):1753-1765. doi: 10.1056/NEJMoa2304192

- JAMA. 2025 May 13;333(18):1608-1617. doi: 10.1001/jama.2025.3169
- BMJ. 2022 Nov 3:379:e071476. doi: 10.1136/bmj-2022-071476

The reason to not adjust for multiplicity the widths of confidence intervals for secondary objectives in the present paper is **based on the following published guidance**: Bender R, Lange S. “Adjusting for multiple testing: when and how?” J Clin Epidemiol. 2001;54(4):343–349. Accordingly, confidence intervals for secondary outcomes are unadjusted for multiplicity and reported p-values are nominal; these results are therefore exploratory and should not be interpreted as confirmatory. Only the primary outcome was analyzed confirmatorily using the prespecified alpha level. Other teams have recently published the results of randomized clinical trials with the same methodology, showing that this is well accepted. For example, the results of the phase III clinical trial with Orforglipton in early type 2 diabetes, reported in the New England Journal of Medicine (Rosenstock et al., N Engl J Med. 2025 Sep 18;393(11):1065-1076. doi: 10.1056/NEJMoa2505669) or the phase II clinical trial with berberine ursodeoxycholate in patients with NASH in Nature communications (Harrison et al., Nat Commun. 2021 Sep 17;12(1):5503. doi: 10.1038/s41467-021-25701-5).

Reviewer #3 (Remarks to the Author):

To the authors,

The randomized controlled trial, “Effects of Totum-63 on fasting plasma glucose in individuals with dysglycemia: a large-scale randomized double-blind placebo-controlled trial.”, conducted by Chavanelle et al. investigated the efficacy of Totum-63 (T63) in improving FPG in subjects with prediabetes or early-stage T2D. After six months, FPG was significantly reduced in both the T63 TID and BID groups compared to placebo. T63 also improved HbA1c, glucose tolerance, body weight, and lipid profile, with the extent of these benefits varying by dosing schedule (BID vs. TID). Although the novel compound is of interest, I may share the following concerns:

- The authors list “no significant change in food habits or physical activity in the 3 months prior” as an inclusion criterion; however, lifestyle adjustments should have been actively implemented and standardized before trial initiation, as is common in trials of glucose-lowering agents. This helps ensure a uniform baseline and allows the drug’s effect to be isolated. Instead, the additional provision of “general lifestyle recommendations” introduces uncontrolled variability from baseline, potentially confounding the observed outcomes.

We thank the reviewer for highlighting the central role of lifestyle in trials evaluating metabolic outcomes. We fully agree that dietary intake and physical activity warrant careful control. In REVERSE-IT, these aspects were handled rigorously, as detailed below.

1) Rationale for the design and alignment with standard of care

Our study enrolled adults with prediabetes or newly diagnosed, untreated early type 2 diabetes populations, for whom initial lifestyle modification is recommended as standard of care (ADA and EASD consensus report, Diabetologia 2022: 65:p1925). Accordingly, standardized lifestyle recommendations were initiated at baseline in all randomized groups (placebo and active), thereby

reflecting routine clinical practice in this setting while preserving internal validity through randomization. We acknowledge that a prerandomization “run-in” to standardize lifestyle can reduce variance; however, our design choices were made to (1) mirror real-world management and (2) ensure uniform counselling across arms at study start. This timing is common practice in high-quality metabolic/obesity RCTs, including trials published in leading journals. For example, a phase-2 RCT in obesity with cardiometabolic endpoints (Jastreboff et al., 2023, NEJM) and an RCT in adults with prediabetes and obesity assessing diabetes risk with liraglutide (Le Roux et al., 2017, The Lancet) implemented lifestyle counselling at randomization.

2) Sample-size assumptions and observed variability

The primary endpoint power calculation used variability estimates from Sirvent et al. (pilot study **without a lifestyle run-in**; Diabetes, Obes Metab. 2022, 24(12):2331), thereby anticipating the variance under our design. Importantly, **the observed variance on the primary endpoint was lower than in that pilot**, supporting adequate power under real-world conditions.

3) Methodological safeguards (eligibility, measurement, and monitoring of lifestyle variables)

- **Eligibility criteria** were chosen to **minimize baseline heterogeneity** and subsequent drift. Specifically, participants were required to have stable weight and no material change in dietary habits or physical activity during the 3 months prior to enrollment; individuals with extreme or unbalanced diets, extreme physical-activity patterns, or use of supplements/functional foods liable to affect metabolic endpoints were excluded (Supplementary Table 1).

- **Lifestyle variables were measured with validated tools and expert oversight**. Diet was assessed using 3-day food diaries (two weekdays and one weekend day) collected before each visit and analyzed by a registered dietitian with Nutrilog software. From these surveys we derived total energy intake; percent energy from fat, carbohydrate, and protein; saturated-fat intake; and alcohol intake (micronutrients were also captured but not used for primary adjustments). Physical activity (PA) was assessed with the IPAQ-SF (7-day recall), capturing type, intensity, average duration, and frequency of main activities, including occupational activity.

- To limit inter- and intragroup variability over time, **adherence to lifestyle recommendations was reinforced and documented at every visit** through structured interviews conducted by trained investigators (or delegates) and via **scheduled telephone follow-ups every 4 weeks between visits** to both reiterate lifestyle guidance and verify changes since the previous assessment.

4) Baseline comparability

At baseline, groups were well balanced for both physical activity and dietary variables. Using standard between-group comparisons, we observed no statistically significant differences for total energy intake, macronutrient distribution (percent energy from fat, carbohydrate, protein), saturated-fat intake, alcohol intake, or for the IPAQ-derived overall physical activity score and its components (type, intensity, duration, frequency, including occupational activity). This baseline homogeneity substantially reduces the risk of lifestyle-related confounding at study outset.

5) Longitudinal consistency and sensitivity analyses

- Across the supplementation period, **no group-by-time interaction was detected for physical activity metrics or dietary variables**; trajectories were comparable between arms. Consistent with our rigorous monitoring, a blinded data-review meeting flagged only 5 participants (0.79%) with

major lifestyle deviations during follow-up. These individuals were excluded from the per-protocol (PP) set (3 in placebo; 2 in active). **PP analyses confirmed the ITT results**, supporting robustness.

- In addition, **prespecified sensitivity analyses adjusting for potential confounders (total energy intake and the overall physical activity score - individually and jointly), left the primary findings unchanged** (and likewise supported the main secondary endpoints). Taken together, the absence of differential lifestyle changes over time, coupled with concordant adjusted and PP results, indicates that the observed product effects are not explained by lifestyle variability.

6) Manuscript revisions (now included in the resubmission)

- Supplementary Methods (Supplementary files, page 1): **Expanded details on how diet and physical activity were assessed** (tools, timing, variables).

- Supplementary Methods (Supplementary files, page 1): **Detailed description of the content, implementation, and reinforcement of lifestyle recommendations**.

- Methods/Statistics (page 7, line 171) & Results (page 9, line 216): Added the **methods and findings for sensitivity analyses adjusted for lifestyle confounders** (total energy intake and overall physical-activity score (individually and jointly)).

- Methods/Statistics (page 7, line 174) & Results (page 9, line 215): Added the **methods and results for the per-protocol (PP) confirmatory analysis**.

- Table 1 (page 20): Added **baseline total energy intake** from dietary surveys.

- Results (page 12, line 290): Reported the **longitudinal evolution of physical-activity and dietary parameters**.

- Supplementary Table 3 (Supp. files, page 9): Provided **changes in overall physical-activity score and total energy intake** at baseline, 3 months, and 6 months.

In summary, while a prerandomization lifestyle run-in can reduce variance, our design reflects standard of care and incorporated key safeguards to isolate the product effect: **standardized counselling at randomization across arms, strict eligibility** ensuring 3-month lifestyle stability, and **validated assessments with reinforced monitoring**. This approach is common in high-quality metabolic RCTs published in leading journals. **Groups were balanced at baseline, no differential lifestyle changes emerged over time, and per-protocol as well as confounder-adjusted sensitivity analyses reproduced the primary findings**. Collectively, these points make lifestyle-related confounding unlikely and support the internal validity and clinical relevance of the observed effect. We have made substantive additions to the Methods and Results sections, which we believe materially improve the manuscript's clarity for readers.

- Although the study showed statistical significance in its primary outcome, the absolute effect size was relatively small (1.9 and 4.25 mg/dL in the TID and BID groups, respectively) and notably lower than the expected target of 10 mg/dL. The sample size was calculated to provide 88% power to detect a 10 mg/dL difference in FPG between T63 TID and placebo at 24 weeks. However, the observed treatment effect was substantially smaller than anticipated. As a result, the study was likely overpowered (underpowered?), and the statistical significance of the findings should be

interpreted with caution, as even clinically marginal differences may reach significance in such settings.

We thank the reviewer for these comments. We are happy to address them below.

1/ Planning assumptions vs. observed data.

The **10 mg/dL target used for sample-size planning came from the only available pilot study** (Sirvent et al., Diabetes Obes Metab, 2022), in which the between-arm contrast **was largely driven by an increase in the small placebo group (n = 13)**, implying **substantial uncertainty around that planning effect and the variance**. In the present trial, **the observed variance of fasting plasma glucose was lower than assumed**, which **preserved adequate statistical precision** for the T63 TID comparison (≈ 80% design-equivalent power) and yielded even greater precision for T63 BID (> 95%). Thus, the present findings should not be dismissed based solely on statistical grounds because the realized effect size was smaller than the planning assumption.

2/ Clinical relevance.

We agree that statistical significance does not automatically imply clinical significance; inference should focus on the effect estimates and their 95% CIs. **In large diabetes-prevention trials such as the Diabetes Prevention Program** (DPP; NEJM 2002 and Maruthur et al., J Gen Intern Med 2013), **early changes in fasting glucose were on the order of ~-3 mg/dL versus placebo in both active arms (metformin or intensive lifestyle)**. Yet **these glycemic shifts co-occurred with substantial long-term reductions in diabetes incidence** (−58% lifestyle; −31% metformin). This illustrates that **short-term mean shifts in FPG of this order can be clinically meaningful** at the population level. In our study, the placebo-adjusted estimates (**T63 TID −3.8 mg/dL**, 95% CI −5.9 to −1.7; **T63 BID −5.5 mg/dL**, 95% CI −9.7 to −1.3) **are of similar or greater magnitude**, with 95% CIs excluding zero. They fall within the range typically observed in credible prevention programs, supporting clinical relevance beyond statistical significance alone. Moreover, clinical relevance should be judged in the context of the totality of outcomes: the product also showed favorable effects on additional parameters (e.g., body weight, other glucose-homeostasis measures, and lipid profile), which further reinforces the potential clinical significance of the results.

For transparency, we have clarified in the Methods that the 10 mg/dL figure was a planning assumption from a small pilot (page 6 line 154).

- The authors state that their results are comparable to those of the meta-analysis by Zhao et al. However, this comparison is only applicable to prediabetic patients, in whom exercise and metformin were associated with FPG reductions of −0.26 mmol/L (about -4.7 mg/dL) and −0.33 mmol/L (about -5.9 mg/dL), respectively. Hence, the treatment effect observed in the exercise alone group was greater than that observed in the present study. Importantly, in the study of Zhao et al., patients with T2D, patients had a reduction of FPG of −33.84 mg/dL and −33.30 mg/dL in the exercise and metformin groups, respectively, thus greatly surpassing the treatment effect noted in the present study.

We thank the reviewer for this constructive comment, which highlights the importance of a precise comparison with the meta-analysis by Zhao et al. (Br J Sports Med, 2024). The reviewer is correct that this comparison deserves further clarification.

First, we respectfully point out that the values quoted by the reviewer for type 2 diabetes (-33.84 and -33.30 mg/dL for exercise and metformin, respectively) do not correspond to those reported in Zhao et al. We believe that this discrepancy may stem from an unintentional mix-up between Table 14.8 (fasting glucose) and Table 14.9 (fasting insulin) in Zhao et al. (page 101 of the supplemental material), which could easily occur inadvertently. Indeed, the fasting-insulin values reported in Table 14.9 (-1.85 µU/mL and -1.88 µU/mL), when multiplied by the usual glucose conversion factor (mmol/L to mg/dL), yield approximately -33.30 and -33.84, respectively. In addition, when comparing effect sizes across studies, it is essential to compare like with like, particularly regarding the analytical approach. Zhao et al. explicitly state **that their main analyses were performed using per-protocol (PP)** data whenever both intention-to-treat (ITT) and PP results were available, and they emphasize that **PP analyses tend to overestimate treatment effects**, while ITT analyses provide more conservative and robust estimates (Zhao et al., page 1457 in the Subgroup analyses and page 1459 in the Limitations of the study). Moreover, Zhao et al. also **demonstrated that baseline fasting plasma glucose (FPG) is a major determinant of the magnitude of the observed reduction** (Zhao et al., page 1457 in the Subgroup analyses), with higher baseline values predicting larger decreases ($\beta = 0.363$, $p < 0.001$, Table 16.2). Taken together, these elements justify a more refined comparison between our results and those of Zhao et al., both in prediabetic and in diabetic participants, as elaborated below.

A/ Prediabetic participants

In our ITT analysis, the placebo-adjusted mean changes in FPG were -3.7 mg/dL in the T63 TID group and -5.2 mg/dL in the T63 BID group, thus averaging approximately -4.5 mg/dL across our two supplemented arms. In Zhao et al., the main estimates (based on PP analysis) showed FPG reductions of -0.26 mmol/L (~-4.7 mg/dL) with exercise and -0.33 mmol/L (~-5.9 mg/dL) with metformin (Zhao et al., page 1455). These are therefore very similar to our values. However, Zhao et al. also reported the results from studies analyzed in ITT only, which are methodologically comparable to our own analysis. Under these ITT-only conditions, the FPG reductions fell to -0.13 mmol/L (~-2.3 mg/dL) for exercise and -0.17 mmol/L (~-3.1 mg/dL) for metformin (Zhao et al., Table 15.2 page 108 in the supplemental material). Hence, **when comparing on an ITT-to-ITT basis, our results show larger reductions in fasting glucose than both exercise and metformin interventions reported by Zhao et al. in prediabetic individuals**.

B/ Type 2 diabetic participants

For type 2 diabetes, in our study the placebo-adjusted mean changes in FPG were -4.3 mg/dL in the T63 TID group and -7.6 mg/dL in the T63 BID group, thus averaging approximately -6 mg/dL across our two supplemented arms. Regarding values reported by Zhao et al., the reviewer's quoted reductions (-33.84 and -33.30 mg/dL) are not consistent with the values reported in the manuscript. In their main analysis (PP), Zhao et al. found average reductions of -0.85 mmol/L (~-15.3 mg/dL) with exercise and -1.52 mmol/L (~-27.4 mg/dL) with metformin (Table 14.8 page 101 in the supplemental material and page 1455 in the Results section of the main manuscript). Moreover, in the ITT-only analyses, the reductions were smaller, -0.45 mmol/L (~-8.1 mg/dL) for exercise and -1.56 mmol/L (~-28.1 mg/dL) for metformin (Zhao et al., Table 15.3 page 109 in the supplemental material). Importantly, **Zhao et al. demonstrated that baseline FPG strongly predicts treatment magnitude** (Table 16.2 page 111 in the supplemental material, $\beta = 0.363$ [0.303–0.424], $p < 0.001$), meaning that for each 1 mmol/L lower baseline FPG, the expected efficiency is ~0.36 mmol/L smaller. Their dataset

indicates average baselines of approximately 8.4 mmol/L (~151 mg/dL) for exercise trials and 10.0 mmol/L (~180 mg/dL) for metformin trials in T2D (Table 9.2 page 72 in the supplemental material). **In our study, baseline FPG values were lower** (146 mg/dL, ~8.1 mmol/L), reflecting an early-stage T2D population. By applying Zhao et al.'s regression coefficient (β = 0.363), **we can estimate the expected metformin- or exercise-induced FPG changes at our baseline levels: -0.85 mmol/L (~-15 mg/dL) for metformin and -0.33 mmol/L (~-6 mg/dL) for exercise**. These adjustments demonstrate that, **at the same baseline FPG and under ITT analysis, the expected effect of exercise (-6 mg/dL) is very close to that observed with T63 in our T2D subgroup**, whereas the expected effect of metformin (-15 mg/dL) remains substantially greater.

Implications for the manuscript:

Thanks to the reviewer, this more detailed examination of the meta-analysis by Zhao et al. allowed us to refine and clarify the comparison with our own study results for both the prediabetic and early T2D participants. In the revised manuscript, we reorganized the Discussion section by separating the analyses for the prediabetic and early type 2 diabetes subpopulations (Page 13, line 325; page 13 line 332; page 14 line 345). For the T2D subgroup, we explicitly highlighted the limitations related to the smaller sample size and the need for caution in interpreting these results. We deliberately chose not to include detailed numerical comparisons with Zhao's meta-analysis for the T2D subgroup because, as explained above, the populations are not directly comparable and any extrapolation of effect sizes to match baseline glycemia would be too speculative for inclusion in the main text. Nevertheless, and as noted by the reviewer, we now clearly state in the revised Discussion that the magnitude of metformin's effect is greater than that of T63 in individuals with type 2 diabetes (Page 14 line 350). This statement is supported both by Zhao's meta-analysis and by direct comparison with the results of the UKPDS trial (UKPDS, The Lancet, 2018), which included participants with baseline fasting glucose levels similar to those in the REVERSE-IT population.

- Among 1,846 subjects screened, only 636 were eligible and randomized. Given that the drug appears to target a broad population, this high screen failure rate raises concerns about the representativeness of the enrolled cohort. Such selective inclusion may limit the external validity and generalizability of the findings—particularly to real-world patients with comorbidities or diabetes-related complications.

Among the 1846 subjects screened, 1210 (65.5%) were classified as screening failures. Importantly, **the majority of exclusions (931 subjects, 77%) were due to glycemic criteria** (FPG or HbA1c values outside the predefined inclusion range). **This high proportion reflects the broad and non-selective recruitment strategy used in our study** (participants were invited to screening without requiring recent glycemic measurements). Consequently, many individuals presented with values outside the predefined prediabetic or early type 2 diabetic range only after formal testing. This explains the numerical proportion of screening failures and **does not indicate an overly restrictive or selective inclusion process**. In fact, the **randomized cohort included both prediabetic individuals and those with early-stage type 2 diabetes** not requiring immediate pharmacological treatment, i.e., the **entire spectrum of metabolic conditions targeted by the investigational product**. Therefore, we believe that **the study population remains fully representative** of the intended target group, and that **this does not compromise the external validity or generalizability of the findings** to real-world individuals within the prediabetes / early T2D continuum.

Minor Comments:

- Please provide a rationale for why the BID regimen group was open-label

The TID regimen (5 g/day in three daily doses) was compared with a matching placebo TID group under a double-blind design, which constituted the confirmatory phase of the trial. The BID regimen (same total daily dose administered in two doses) **was included as an exploratory arm to preliminarily assess the effect of a simplified dosing schedule on glycemic outcomes**.

Maintaining blinding for this exploratory arm would have required the creation of a separate placebo group with a BID dosing schedule, leading to a substantially more complex and resource-intensive study design, essentially equivalent to conducting a second independent trial. For this reason, and given the exploratory nature of the BID assessment, the decision was made to conduct this arm in open-label conditions, while preserving the full double-blind design and statistical integrity of the main TID vs placebo comparison.

Following the reviewer's comment, we have added a clarification in the Methods section of the revised manuscript (Page 5 line 113) to explicitly state the rationale for conducting the BID arm under open-label conditions.

- Describe if T63 is expected to have any drug-drug interactions with other substances

We thank the reviewer for this question regarding potential drug-drug interactions.

T63 is a blend of five food-grade botanical extracts: olive leaf (Olea europaea L.), bilberry fruit (Vaccinium myrtillus L.), artichoke leaf (Cynara cardunculus L. syn. C. scolymus), chrysanthellum (Chrysanthellum indicum subsp. afroamericanum), and black pepper (Piper nigrum L.). **All five plants are authorized for use in foods and food supplements within the European Union and have a long history of safe traditional consumption in Europe**.

According to the European Medicines Agency (EMA) / Committee on Herbal Medicinal Products (HMPC) monographs, no interactions have been reported for artichoke leaf, bilberry fruit, or olive leaf (Section 4.5 "Interactions with other medicinal products and other forms of interaction" of the corresponding monographs explicitly states "none reported"). For chrysanthellum and black pepper, no EMA/HMPC monographs are available; however, **both species are listed by the French competent authority (DGCCRF) and the European authority (EFSA) among botanicals authorized for use in food supplements, and no regulatory warnings or restrictions related to drug interactions are mentioned for either**.

Taken together, these official European regulatory documents indicate that no clinically relevant drug-drug interactions are known or recognized for the botanical ingredients included in T63. Their longstanding traditional dietary use and regulatory acceptance as food-grade botanicals further support the absence of expected pharmacological interactions at the doses used in this study.

- Consider providing a more informative table description for Table 2

We thank the reviewer for this helpful suggestion. We have revised the legend of Table 2 (Page 25) to provide a more detailed description of the table content and methodology, including the definition and assessment of product compliance across study visits.

The formatting of Table 4 is unclear, as the asterisk symbols (e.g., *) do not consistently correspond to the p-values listed below. Please clarify their meaning in the table legend.

We thank the reviewer for this observation. The table presents two distinct sets of statistical comparisons: 1/ intergroup comparisons versus placebo, for which p-values are reported numerically, and 2/ intragroup comparisons versus baseline, indicated by asterisks (*p<0.05, **p<0.01, ***p<0.001, ****p<0.0001). To avoid confusion, we have slightly clarified the table legend to make this distinction more explicit (Page 25).

Additional comment

- The press release information found under https://investisseurs.valbiotis.com/en/totum-63/ references this study and published claims not justified by the present data. For example, it states that T63 significantly reduces fasting blood glucose (-8.1 mg/dl), 2-hour blood glucose (-21.9 mg/dl), glycated hemoglobin (-0.18%) and the HOMA-IR insulin resistance score (-1.04 pts), compared with placebo. group and placebo. The actual reduction from baseline within the T63 group is substantially smaller, and should be used for a proper interpretation of the treatment effect. Furthermore, the authors claim a significantly reduced progression towards type 2 diabetes, an outcome not investigated in the present study (only deterioration in glycemic status was assessed). A final example is found in the claim that "The study confirms TOTUM63's excellent safety profile, with no risk of hypoglycemia.". Again, the present study did not investigate the association between T63 and risk of hypoglycemia. Finally, the product is labeled as a dietary supplement, in which it is strictly prohibited to claim disease prevention or treatment. Such claims are reserved exclusively for drugs that have undergone rigorous FDA review and approval.

We acknowledge that the phrasing used in the press release could, in retrospect, have been more precisely formulated, and we appreciate the reviewer's comment, which allows us to clarify these aspects here. However, we respectfully argue that no figures were incorrect or inconsistent with the study data, or that the communication raised potential regulatory issues. We are pleased to provide full clarification on all the points raised.

First, regarding the numerical values cited (e.g., fasting plasma glucose -8.1 mg/dL, 2-hour glucose -21.9 mg/dL, HbA1c -0.18%, HOMA-IR -1.04), these figures are fully consistent with the study data and **correspond to the unadjusted mean differences between the T63 BID and placebo groups**. The BID regimen was chosen for external communication because it represents the intended real-world dosing. This was explicitly stated in the press release, which clearly indicated that the numerical values reported referred to the BID regimen group. **For example, for fasting plasma glucose, the manuscript reports a mean increase of +3.86 mg/dL in the placebo group and a mean decrease of -4.25 mg/dL in the T63 BID group** (page 9, line 226-228), yielding an unadjusted "raw" difference of -8.11 mg/dL, precisely the value communicated in the press release. These same mean values for the

placebo (+3.86 mg/dL) and T63 BID groups (-4.25 mg/dL) **are explicitly presented in the press release itself**, showing both the increase in the placebo group and the decrease in the active group. This clearly demonstrates that the reported difference of −8.11 mg/dL reflects the combined effect of both directions of change and **was communicated without ambiguity or intent to overstate the treatment effect**. In the manuscript, the same endpoints **are also presented using adjusted least-squares mean differences derived from the statistical model, which are more appropriate for scientific reporting but necessarily yield numerically different estimates.** Thus, the **press release figures are accurate, transparent, and entirely aligned with the study dataset**.

Secondly, regarding the statement referring to a "reduction in progression toward type 2 diabetes," we acknowledge that this wording may appear somewhat imprecise. However, we would like to provide additional clarification here to explain the basis for this communication and its consistency with the study data. In the present study, deterioration of glycemic status was prespecified and assessed as an exploratory endpoint in the overall population. Within the prediabetic subgroup, such deterioration specifically corresponds to progression to type 2 diabetes, which is the basis for the wording used in the press release. While the subgroup-specific calculation of deterioration in prediabetic participants was performed and was consistent with this interpretation, it was not included in the manuscript because it was exploratory and, given space constraints and the need to prioritize primary and key secondary outcomes, we could not present all analyses across all subpopulations.

Below is presented the graph specifically for the subgroup with prediabetes, showing a reduction of more than 40% in the BID group compared with placebo.

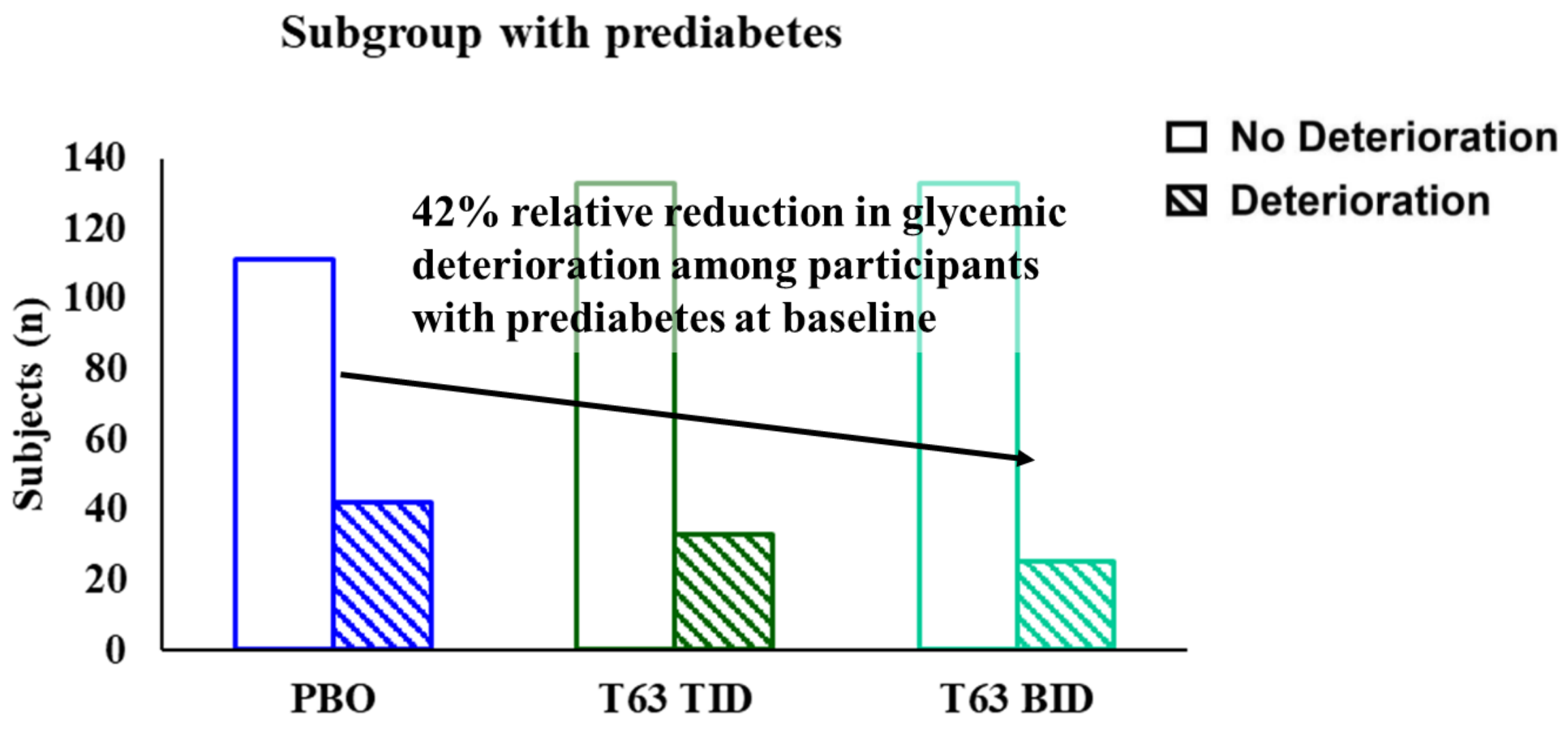

We also acknowledge that the definition of glycemic deterioration used in our exploratory analyses (transitioning from an FPG value < 1.26 g/L to ≥ 1.26 g/L) does not fully correspond to the formal diagnostic criteria for type 2 diabetes. According to standard definitions, type 2 diabetes is established by **either two consecutive FPG measurements ≥ 1.26 g/L or a postprandial glucose value ≥ 2 g/L.** Therefore, within the prediabetic subgroup, we additionally assessed the number of participants who, at the end of the study, actually met these biological criteria precisely defining type 2 diabetes (i.e., two consecutive FPG ≥ 1.26 g/L or postprandial glucose ≥ 2 g/L). The results, presented below, showed a **relative reduction in the proportion of participants meeting the biological criteria for type 2 diabetes of 58% in the TID group and 76% in the BID group compared**

with placebo. This demonstrates that applying the stricter diagnostic criteria would have led to even greater relative reductions than the ~40% decrease reported in the press release, further confirming that, while this initial public communication could perhaps have been more technically explicit, it did not in any way overstate the findings.

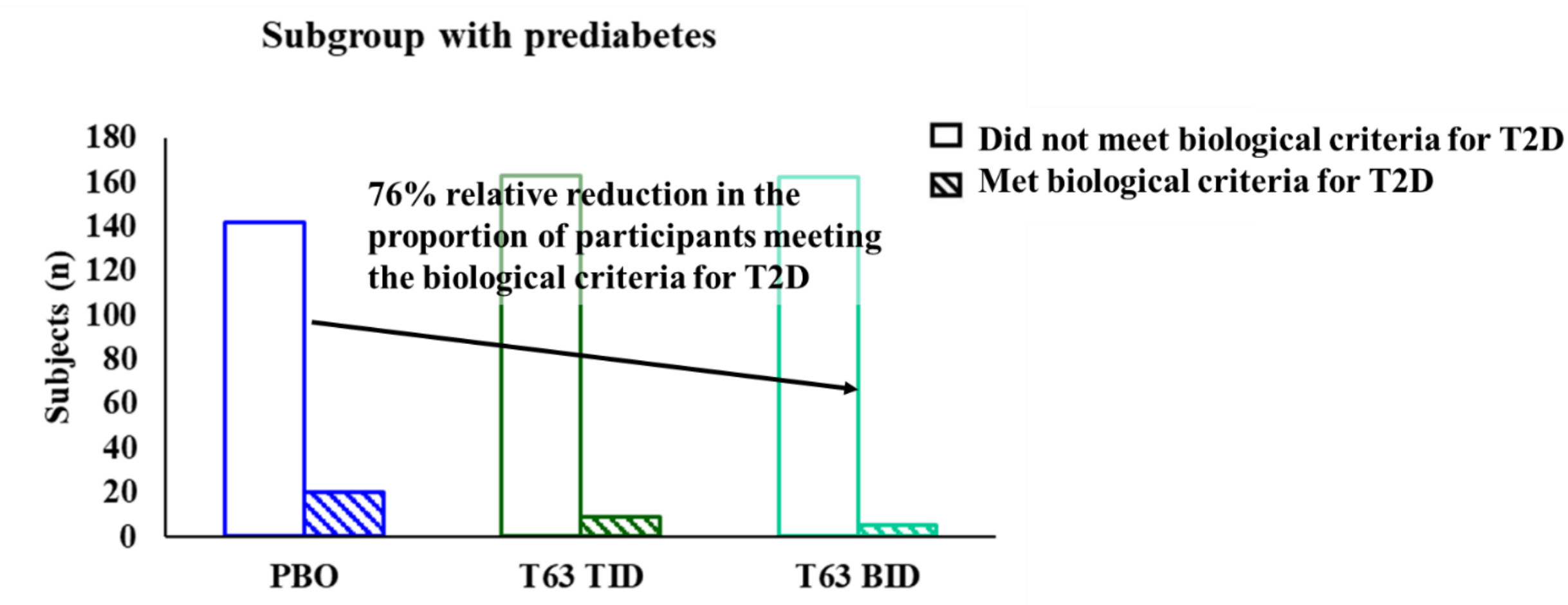

Thirdly, regarding hypoglycemia, we acknowledge that hypoglycemia risk was not a prespecified endpoint in this trial. The press-release wording was intended to reflect that no hypoglycemic events were reported in the safety dataset. However, we agree that this phrasing could have been clearer and should not be interpreted as a formal assessment of hypoglycemia risk. In retrospect, a more precise formulation would have explicitly stated that no hypoglycemic events were observed in the study rather than implying any broader conclusion about risk.

Finally, we wish to emphasize that the press release fully complies with EU regulatory requirements. It communicates the results of a clinical study on TOTUM-63 as an **active ingredient**, not as a marketed food supplement. Under Regulation (EC) No 1924/2006, such scientific reporting is **distinct from health claims applicable to finished consumer products**, and it is therefore entirely permissible to reference disease-related outcomes in the context of research disclosure. The same regulatory principle applies in the United States, where communication of scientific results concerning an investigational active ingredient is not subject to the restrictions governing labeling or marketing claims for finished dietary supplements or drugs.

Reviewer #4 (Remarks to the Author):

I co-reviewed this manuscript with one of the reviewers who provided the listed reports. This is part of the Nature Communications initiative to facilitate training in peer review and to provide appropriate recognition for Early Career Researchers who co-review manuscripts.

Reviewer #5 (Remarks to the Author):

Effects of Totum-63 on fasting plasma glucose in individuals with dysglycemia: a large- scale randomized double-blind placebo-controlled trial by V Chavanelle et al.

A multicentre RCT of a plant-based product (3-times a day) was conducted with 6 months intervention in adults with pre-diabetes or Type 2 diabetes was conducted. Individuals from 37

seven European countries with FPG between 110-220 mg/dL were randomized to receive 38 placebo (PBO, n=210), T63 (5 g/d) distributed thrice daily (T63 TID, n=212) or twice daily 39 (T63 BID, open-label arm, n=214) over six months. The primary outcome was the FPG change in T63 TID compared to PBO in the intention-to-treat population. Secondary assessments included changes in diabetes biomarkers, lipid profile, anthropometric measurements and subpopulation analyses based on glycemia. After 6-months, FPG was significantly reduced in the T63 TID group (PBO-adjusted mixed model estimated difference, PBO-AD=-44 3.8 mg/dL, 95% CI: [-5.9; -1.7], p=0.0149) and the T63 BID group (PBO-AD=-5.5 mg/dL, 45 95% CI: [-9.7; -1.3] p<0.001). T63 also improved HbA1c, glucose tolerance, body weight and lipids, with magnitude of benefits differing based on the distribution pattern. Subpopulation analyses showed efficacy of T63 in subjects with prediabetes.

Comments: Pre-diabetes and Type 2 diabetes are pandemic and a major cause of morbidity, premature mortality and high socioeconomic costs. Treatments to prevent progression of dysglycaemia are needed.

There was no run-in period with lifestyle changes only, and this and the Hawthorne effect could contribute to the benefits noted, even though there is a placebo group.

We thank the reviewer for this valuable comment, which is consistent with a similar remark raised by Reviewer N3 regarding the absence of a prerandomization lifestyle run-in and the potential influence of behavioral effects such as the Hawthorne effect. We have addressed this issue comprehensively below.

We fully agree that dietary intake and physical activity warrant careful control. In REVERSE-IT, these aspects were handled rigorously, as detailed below.

1) Rationale for the design and alignment with standard of care

Our study enrolled adults with prediabetes or newly diagnosed, untreated early type 2 diabetes populations, for whom initial lifestyle modification is recommended as standard of care (ADA and EASD consensus report, Diabetologia 2022: 65:p1925). Accordingly, standardized lifestyle recommendations were initiated at baseline in all randomized groups (placebo and active), thereby reflecting routine clinical practice in this setting while preserving internal validity through randomization. We acknowledge that a prerandomization "run-in" to standardize lifestyle can reduce variance; however, our design choices were made to (1) mirror real-world management and (2) ensure uniform counselling across arms at study start. **This timing is common practice in high-quality metabolic/obesity RCTs**, including trials published in leading journals. For example, a phase-2 RCT in obesity with cardiometabolic endpoints (Jastreboff et al., 2023, NEJM) and an RCT in adults with prediabetes and obesity assessing diabetes risk with liraglutide (Le Roux et al., 2017, The Lancet) implemented lifestyle counselling at randomization.

2) Sample-size assumptions and observed variability

The primary endpoint power calculation used variability estimates from Sirvent et al. (pilot study **without a lifestyle run-in**; Diabetes, Obes Metab. 2022, 24(12):2331), thereby anticipating the variance under our design. Importantly, **the observed variance on the primary endpoint was lower than in that pilot**, supporting adequate power under real-world conditions.

3) Methodological safeguards (eligibility, measurement, and monitoring of lifestyle variables)

- **Eligibility criteria** were chosen to **minimize baseline heterogeneity** and subsequent drift. Specifically, participants were required to have stable weight and no material change in dietary habits or physical activity during the 3 months prior to enrollment; individuals with extreme or unbalanced diets, extreme physical-activity patterns, or use of supplements/functional foods liable to affect metabolic endpoints were excluded (Supplementary Table 1).

- **Lifestyle variables were measured with validated tools and expert oversight**. Diet was assessed using 3-day food diaries (two weekdays and one weekend day) collected before each visit and analyzed by a registered dietitian with Nutrilog software. From these surveys we derived total energy intake; percent energy from fat, carbohydrate, and protein; saturated-fat intake; and alcohol intake (micronutrients were also captured but not used for primary adjustments). Physical activity (PA) was assessed with the IPAQ-SF (7-day recall), capturing type, intensity, average duration, and frequency of main activities, including occupational activity.

- To limit inter- and intragroup variability over time, **adherence to lifestyle recommendations was reinforced and documented at every visit** through structured interviews conducted by trained investigators (or delegates) and via **scheduled telephone follow-ups every 4 weeks between visits** to both reiterate lifestyle guidance and verify changes since the previous assessment.

4) Baseline comparability

At baseline, groups were well balanced for both physical activity and dietary variables. Using standard between-group comparisons, we observed no statistically significant differences for total energy intake, macronutrient distribution (percent energy from fat, carbohydrate, protein), saturated-fat intake, alcohol intake, or for the IPAQ-derived overall physical activity score and its components (type, intensity, duration, frequency, including occupational activity). This baseline homogeneity substantially reduces the risk of lifestyle-related confounding at study outset.

5) Longitudinal consistency and sensitivity analyses

- Across the supplementation period, **no group-by-time interaction was detected for physical activity metrics or dietary variables**; trajectories were comparable between arms. Consistent with our rigorous monitoring, a blinded data-review meeting flagged only 5 participants (0.79%) with major lifestyle deviations during follow-up. These individuals were excluded from the per-protocol (PP) set (3 in placebo; 2 in active). **PP analyses confirmed the ITT results**, supporting robustness.

- In addition, **prespecified sensitivity analyses adjusting for potential confounders (total energy intake and the overall physical activity score (individually and jointly)), left the primary findings unchanged** (and likewise supported the main secondary endpoints). Taken together, the absence of differential lifestyle changes over time, coupled with concordant adjusted and PP results, indicates that the observed product effects are not explained by lifestyle variability.

6) Manuscript revisions (now included in the resubmission)

- Supplementary Methods (Supplementary files, page 1): **Expanded details on how diet and physical activity were measured** (tools, timing, variables).

- Supplementary Methods (Supplementary files, page 1): **Detailed description of the content, implementation, and reinforcement of lifestyle recommendations**.

- Methods/Statistics (page 7, line 171) & Results (page 9, line 216): Added the **methods and findings for sensitivity analyses adjusted for lifestyle confounders** (total energy intake and overall physical-activity score (individually and jointly)).

- Methods/Statistics (page 7, line 174) & Results (page 9, line 215): Added the **methods and results for the per-protocol (PP) confirmatory analysis**.

- Table 1 (page 20): Added **baseline total energy intake** from dietary surveys.

- Results (page 12, line 290): Reported the **longitudinal evolution of physical-activity and dietary parameters**.

- Supplementary Table 3 (Supp. files, page 9): Provided **changes in overall physical-activity score and total energy intake** at baseline, 3 months, and 6 months.

In summary, while a prerandomization lifestyle run-in can reduce variance, our design reflects standard of care and incorporated key safeguards to isolate the product effect: **standardized counselling at randomization across arms, strict eligibility** ensuring 3-month lifestyle stability, and **validated assessments with reinforced monitoring**. This approach is common in high-quality metabolic RCTs published in leading journals. **Groups were balanced at baseline, no differential lifestyle changes emerged over time, and per-protocol as well as confounder-adjusted sensitivity analyses reproduced the primary findings**. Collectively, these points make lifestyle-related confounding unlikely and support the internal validity and clinical relevance of the observed effect. We have made substantive additions to the Methods and Results sections, which we believe materially improve the manuscript's clarity for readers.

Plant extracts often have an odour or taste, and some may change urine colour. Was the active drug and placebo identical in smell, taste and appearance and was there any urine or faeces colour change with the active drugs.

We thank the reviewer for this relevant question. The placebo and T63 capsules **were identical in appearance, odor, and taste**, ensuring that participants, investigators, and care providers remained blinded to treatment allocation throughout the study. Both formulations were encapsulated in the **same opaque capsules**, preventing any visual distinction of the powder content. No participant or investigator reported any detectable difference in smell or taste between capsules during follow-up.

Furthermore, there were **no observed or reported changes in urine color or stool appearance** in participants receiving T63 compared with placebo.

To address this point, we have clarified the manuscript accordingly (see revised sentence in the Methods section, page 5 line 115).

What was the time between blood being drawn and separated, as beyond a certain time the plasma or serum glucose level drops due to cellular utilisation?

We thank the reviewer for this important methodological point. All blood samples for fasting and post-load glucose measurements were collected under standardized conditions, identical across all study sites and in accordance with the central laboratory's Standard Operating Procedures (SOPs). Blood was drawn into fluoride/oxalate tubes to inhibit glycolysis and centrifuged within 30 min of

collection (2200 g, 15 min, 20 °C ± 2). Plasma was then aliquoted and stored at −80 °C until analysis. This procedure complies with current recommendations (Sacks et al., Clin Chem 2023) to minimize preanalytical glucose decline due to cellular utilization.

We have added this methodological clarification in the supplementary files (Page 2).

Reference:

Sacks et al. (2023): Guidelines and Recommendations for Laboratory Analysis in the Diagnosis and Management of Diabetes Mellitus. Clinical Chemistry 69:8 p.808–868.

Does T63 affect any of the assays in particular, glucose, HbA1c and insulin? Tests such as assays without and with the addition of T63 to blood are of interest.

We did not perform dedicated in-vitro “spike-in” interference tests with T63. However, based on the chemistry of its constituents, the validated assay methods used, the manufacturers’ documented interference profiles, and our internal quality-control monitoring, analytical interference by T63 is considered unlikely for fasting glucose, HbA1c, or insulin.

What we measured and why interference is unlikely:

Glucose: measured by a central-laboratory hexokinase method (GLUC3, Roche Diagnostics; IFCC-traceable), which quantifies NADPH formation at 340 nm. The hexokinase method is well standardized, widely used in accredited laboratories, and shows low analytical imprecision and high agreement across methods, supporting its robustness and low susceptibility to analytical interference (Sacks et al., Clin Chem 2023). Known interferents include hemolysis, icterus, and lipemia (monitored via HIL indices). According to the manufacturer, no relevant interference was found at therapeutic concentrations for a wide panel of common drugs. T63 contains dietary polyphenols (oleuropein metabolites, chlorogenic/caffeoylquinic acids, anthocyanins, flavonoids…), and piperine that, at physiological post-dose plasma concentrations (low µM/nM and largely conjugated), are not listed as interferents for hexokinase assays.

HbA1c: measured by an immunoturbidimetric/photometric assay (A1C-3, Roche Diagnostics), IFCC-traceable and NGSP/DCCT-aligned. No significant interference was observed up to bilirubin 60 mg/dL or Intralipid 600 mg/dL, nor with common drugs at therapeutic levels. Dietary polyphenols are not listed as interferents.

Insulin: measured by electrochemiluminescent immunoassay (Elecsys, Roche Diagnostics; standardized to the WHO 1st IRP 66/304). According to the manufacturer, potential interferences include hemolysis and high-dose biotin (> 5 mg/day; sampling ≥ 8 h post-dose). No relevant interference was observed up to bilirubin 90 mg/dL, Intralipid 1800 mg/dL, or rheumatoid factor 1200 IU/mL, while acetylcysteine at therapeutic levels can depress measured insulin. Only rare effects from heterophile or anti-reagent antibodies have been described; a high-dose hook effect was not observed up to 20 000 µU/mL. Plant-derived small molecules in T63 are not antigenically related and are not listed as interferents by the manufacturer.

Summary:

Across all visits, **no analyzer flags or out-of-range HIL** (hemolysis, icterus, lipemia) **indices attributable to T63 were observed,** and **the frequency of routine QC flags did not differ between T63 and placebo**. If T63 (or its metabolites) interfered with any of the assays, one would expect visit-independent, assay-specific artifacts, such as systematic offsets or analyzer flags, confined to the active arm. No such patterns were observed: **baseline values were comparable across arms, no analyzer or QC flags were detected, and no assay-specific drift or inconsistency emerged over time**. Together with the manufacturers' interference listings for these assays, these findings argue strongly against any T63-related analytical interference for fasting glucose, HbA1c, or insulin.

Reference:

Sacks et al. (2023): Guidelines and Recommendations for Laboratory Analysis in the Diagnosis and Management of Diabetes Mellitus. Clinical Chemistry 69:8 p.808–868.

Was the HbA1c assay DCCT aligned?

Yes. HbA1c was measured using an immunoturbidimetric/photometric assay (A1C-3, Roche Diagnostics). **This method is standardized against the approved IFCC reference method and results are traceable to DCCT/NGSP** (NGSP-aligned).

What were the intra and inter-assay CVs for each of the assays, and were all samples from a participant on the same run?

We thank the reviewer for this question. All biochemical assays were performed in the central laboratory (Biofortis Innovation Services, France) using Roche Diagnostics platforms and reagents according to the manufacturer's specifications. The intra-assay (repeatability) and inter-assay (reproducibility) coefficients of variation (CVs) for fasting plasma glucose, HbA1c, and insulin measurements are presented in the table below. All values were within the expected analytical performance ranges, confirming excellent precision and assay reliability. These coefficients of variation are now provided in Supplementary Table 5 (Supplementary files, page 11).

Analyte	Instrument / Method	Control level	Intra-assay CV (%)	Inter-assay CV (%)
**Fasting plasma glucose**	Cobas Integra 400+, hexokinase (Roche Diagnostics)	5.56 mmol/L	1.08	2.7
		13.46 mmol/L	0.26	2.6
**HbA1c**	A1C-3 immunoturbidimetric assay (Roche Diagnostics)	5.59%	0.83	0.62

Analyte	Instrument / Method	Control level	Intra-assay CV (%)	Inter-assay CV (%)
		10.67%	1.24	1.94
**Insulin**	Elecsys electrochemiluminescent immunoassay (Roche Diagnostics)	19.6 µIU/mL	1.19	0.8
		96 µIU/mL	1.54	1.0

Samples were analyzed in batches as they were received by the central laboratory, rather than by participant. There were no specific instructions to process all time points from the same participant within a single analytical run. This approach was chosen because, according to the study protocol, investigators were required to review each participant's laboratory results between visits to identify any potential worsening of glycemic status that could require medical attention or study discontinuation. Consequently, samples were processed sequentially after each visit to ensure timely reporting of results, in compliance with the study's safety monitoring procedures.

Continuous Glucose Monitoring (CGM) metrics would have been very valuable in the glucose profiling of the study subjects. Why was CGM not used to assess glycaemia?

We agree that Continuous Glucose Monitoring (CGM) could provide valuable insights into glycemic variability. However, according to current laboratory and clinical recommendations (Sacks et al., Clin Chem 2023), the use of continuous glucose monitoring is mainly indicated for insulin-treated individuals with diabetes who are not meeting glycemic targets, rather than for non-insulin-treated or prediabetic populations. When the protocol was finalized (study initiated in 2020), CGM use and targets were primarily defined for insulin-treated diabetes, and trial-level standardization of CGM metrics (such as time-in-range or glycemic variability) in non-insulin-treated or prediabetic populations was still limited. In addition, CGM implementation would have introduced logistical complexity, additional costs, and potential behavioral modification (e.g., lifestyle adjustments in response to real-time glucose feedback), which could have affected internal validity. Therefore, we relied on well-established, standardized laboratory endpoints (fasting plasma glucose, 2-h OGTT glucose, and HbA1c), which remain the diagnostic and regulatory gold standards for assessing glycemic status in non-insulin-treated individuals. We also acknowledge that CGM could provide additional insights into daily glycemic variability and postprandial excursions beyond the standardized laboratory endpoints used in this study. This point has been added to the limitations section of the revised manuscript (page 16, line 390).

Reference:

Sacks et al. (2023): Guidelines and Recommendations for Laboratory Analysis in the Diagnosis and Management of Diabetes Mellitus. Clinical Chemistry 69:8 p.808–868.

The adverse events are described by severity, not by what they were. Please do so. What type of events were the ones that continued till trial end?

We thank the reviewer for this valuable comment. In accordance with this suggestion, we have added a detailed description of treatment-emergent adverse events (TEAEs) by System Organ Class (SOC), presented in the Supplementary Materials (Supplementary Table 4, page 10).

Moreover, 79 adverse events were still ongoing at the time of study completion, with no significant difference between the three treatment groups (28 in the PLA, 33 in the T63 BID and 18 in the T63 TID). Among these events, 96% were of mild to moderate intensity, and 22% were considered possibly related to the investigational product. Regarding the distribution by physiological system, the events are described in the table below, with the most frequent being gastrointestinal (29.1%) and musculoskeletal/rheumatologic (25.3%).

**Treatment-emergent adverse events ongoing at the end of the study**		
**Body system**	**n**	**%**
Cardiovascular	4	5.1
Dermatological	7	8.9
Endocrine / Hematologic	4	5.1
ENT / Ocular	3	3.8
Gastro-intestinal	23	29.1
Gynecological	1	1.3
Immunological	1	1.3
Locomotor / Rheumatologic	20	25.3
Metabolic	5	6.3
Nephro-urological	4	5.1
Neurologic / Psychiatric	1	1.3
Respiratory	2	2.5
Other	4	5.1
**Total**	**79**	**100**
